# Current Research Trends and Perspectives on Solid-State Nanomaterials in Hydrogen Storage

**DOI:** 10.34133/2021/3750689

**Published:** 2021-01-23

**Authors:** Jie Zheng, Chen-Gang Wang, Hui Zhou, Enyi Ye, Jianwei Xu, Zibiao Li, Xian Jun Loh

**Affiliations:** Institute of Materials Research and Engineering, ASTAR (Agency for Science Technology and Research), 2 Fusionopolis Way, Innovis, #08-03, Singapore, Singapore 138634

## Abstract

Hydrogen energy, with environment amicable, renewable, efficiency, and cost-effective advantages, is the future mainstream substitution of fossil-based fuel. However, the extremely low volumetric density gives rise to the main challenge in hydrogen storage, and therefore, exploring effective storage techniques is key hurdles that need to be crossed to accomplish the sustainable hydrogen economy. Hydrogen physically or chemically stored into nanomaterials in the solid-state is a desirable prospect for effective large-scale hydrogen storage, which has exhibited great potentials for applications in both reversible onboard storage and regenerable off-board storage applications. Its attractive points include safe, compact, light, reversibility, and efficiently produce sufficient pure hydrogen fuel under the mild condition. This review comprehensively gathers the state-of-art solid-state hydrogen storage technologies using nanostructured materials, involving nanoporous carbon materials, metal-organic frameworks, covalent organic frameworks, porous aromatic frameworks, nanoporous organic polymers, and nanoscale hydrides. It describes significant advances achieved so far, and main barriers need to be surmounted to approach practical applications, as well as offers a perspective for sustainable energy research.

## 1. Introduction

Energy serves as the only universal impetus that drives virtually all social and individual activities, such as transportation, residential electricity generation, and commercial applications [1–6]. Because of the rapid growth of the global population (expected to reach 9.7 billion by 2050) and human consumption, the energy demand is going to be continually increasing. Currently, traditional nonrenewable fossil-based fuels—petroleum, coal, and natural gas—afford more than 80% of the global energy. Hence, an energy crisis is inevitable if we continue to consume fossil fuels unscrupulously. As a preliminary speculated, fossil fuels reserve will out of stock shortly, petroleum (40 years), natural gas (60 years), and coal (156 years), for example [6]. Moreover, the greenhouse gases and other pollutants released by the consumption of fossil-based fuel cause serious havoc to the plant, environment, and climate [7–9]. The period of the explosion demand for clean, sustainable, and renewable energies is already come and will continue to last in the next several decades.

Hydrogen is regarded as a nonpolluting, abundant, efficient, and low-cost energy vector for a variety of applications, including stationary power supply, distribution, and diverse mobile hydrogen-fueled platforms. Because of the highest gravimetric energy density (120 MJ/kg), zero emissions of greenhouse gases, and water as the only exhaust product at conversion to energy, hydrogen serves as an ideal long-term solution to energy-related environmental problems [10–17].

Molecular hydrogen (H_2_) can be directly produced from both renewable and nonrenewable sources and has been widely studied in different articles [18–26]. Currently, a variety of nonrenewable sources, such as natural gas, naphtha, heavy oil, and coal, have been used to generate H_2_. The most widely used technique to produce hydrogen in the industry is the steam reforming using fossil fuels [18, 19]. It is a commercially mature technology that can be performed with high efficiency at a low cost. For instance, the hydrogen generation using steam methane reforming can get high efficiency in the range of 65 % to 75 % [20]. However, the steam reforming process is complex and along with the emission of greenhouse gasses such as carbon monoxide and carbon dioxide. Other hydrogen production technologies through reactions with hydrocarbon compounds are including catalytic partial oxidation, autothermal reforming, gasification of coal, and methane decomposition and aromatization. With the development of hydrogen technology, the renewable energy-based processes of hydrogen production, such as solar photochemical and photobiological water decomposition, water electrolysis, and biomass-conversion, have been developed [21–25]. These methods are eco-friendly and high efficiency; however, due to the high cost, they are unable to be the technology of choice for the massive production of H_2_ so far.

As a substitution of fossil fuel, hydrogen energy can bring enormous benefits. However, vital technological and economic challenges need to be surmounted to achieve a sustainable hydrogen economy development. Foremost, among these obstacles is the lack of suitable hydrogen storage approaches. Despite the largest gravimetric energy density, hydrogen has a poor volumetric energy density (0.01 MJ/L for hydrogen vs. 32 MJ/L for gasoline at 0°C and 1 atm), hampering it in large-scale storing at mild condition [15]. The U.S. Department of Energy (DOE) sets the hydrogen capacity targets for onboard storage identifies the significance of both gravimetric and volumetric capacity, where the gravimetric and volumetric capacity means the quantity of hydrogen gas contained in a given weight and volume of the storage system, respectively (Table 1) [14]. The ultimate onboard hydrogen storage goal is 0.065 kg H_2_/kg system and 0.050 kg/L for gravimetric and volumetric, respectively. Typically, a hydrogen storage system contains not only reactant materials but also tanks, compressors, valves, piping, insulation, and other hardware, which comprise a significant proportion of the overall cost. Compare with the pure material storage capacity, the system storage capacity drops significantly as a need to account for all associated components mentioned above. Accordingly, the hydrogen storage material with a high capacity (≥10 wt% hydrogen) and good reversibility (≥1500 cycles) is intensively desired. Additional requirements for an ideal hydrogen storage medium include fast hydrogen uptake/release rate, mild operation, and delivery condition, as well as low cost.

Though H_2_ exhibits various advantages over other energy fuels, it is considered a dangerous fuel mainly because it is a highly combustible gas with a flammability range from 4% to 75% vol in the air and may cause an explosion in the presence of spark, heat, or even sunlight. H_2_ is colorless and odorless, and therefore, confronting to discover in case of leakages. Moreover, own to the density as low as 0.0899 g/L at standard temperature and pressure (STP, 0°C and 1 atm), the storage of hydrogen with conventional approaches, such as compression and liquefaction, need high pressure, and/or very low temperature, resulting in increased leaking risks, and even an explosive if the leakage occurs in a confined surrounding. Hence, safety aspects should be accurately evaluated and taken into account during the hydrogen storage, transportation, and utilization, to avoid hydrogen-related accidents (such as the Hindenburg fire in 1937 and the hydrogen explosion in Fukushima nuclear plant in 2011) happen again.

**Figure 1:**
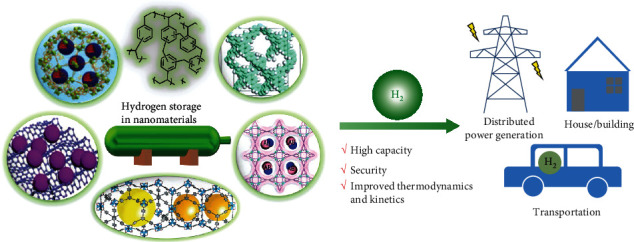
Schematic illustration showing the hydrogen storage in nanomaterials and its sustainable applications. Reproducing from ref ([12, 13, 27–29], and [30]) with permission.

Throughout the past two decades, a great fundamental advancement in the hydrogen technique has been witnessed, particularly in the hydrogen storage [10–17]. The traditional hydrogen storage approach is characterized by physically increase hydrogen gas density using high pressure or extremely low temperature, resulting in the cost and security concerns. Additionally, converting hydrogen into liquid hydrogen-rich molecules, such as formic acid, methanol, ammonia, and liquid organic hydrogen carriers, is also widely explored for hydrogen storage. However, these liquid molecules suffer from relatively low hydrogen capacity, intricate hydrogenation and dehydrogenation reactions, and complicated purification processes. In contrast, physical or chemical storing hydrogen into nanomaterials in the solid-state is a competent and practical alternative (Figure 1) [10–13]. The solid-state hydrogen storage exhibits high hydrogen content, safe, easy for handling, transportation, and tradable.

In this review, we aim to comprehensive summarize the up-to-date solid-state hydrogen storage systems and reveal the related universal rules for hydrogen energy in practical applications. In Section 2, different storage nanomaterials are collated systematically, including nanoporous carbon materials, metal-organic frameworks (MOFs), covalent organic frameworks (COFs), porous aromatic frameworks (PAFs), nanoporous organic polymers, and nanoscale hydrides. Special attention is given to the important roles of nanoscale hydrides for their conspicuous improvement in the hydrogen storage performance. In solid hydrogen storage systems, hydrogen release is triggered by the catalytic dehydrogenation or thermal stimulation of the carriers. In the third part, we discuss the applications of hydrogen fuel in both stationary and mobile platforms. This review concludes by offering developing strategies for endeavouring to improve hydrogen storage performance toward sustainable and economical applications.

## 2. Hydrogen Storage Approaches

Hydrogen gas is the lightest gas (density = 0.0899 g/L at STP) in nature. It contains excellent gravimetric energy storage density (120 MJ/kg) and extremely low volumetric energy density (0.01 MJ/L). Therefore, how to efficiently store this unusual energy carrier is the persistent agonizing thing to wholly develop hydrogen technology. It is believed that the settlement of this problem could lead to significant progress in hydrogen technology. Besides, in a practical application, the hydrogen storage system needs to account for the operation, storage, and transportation efficiency, security, and cost issues. In this section, different hydrogen storage systems with benefits and drawbacks are comprehensively summarized.

### 2.1. Conventional Hydrogen Storage Systems

#### 2.1.1. Compressed Gas

Hydrogen gas pressurized into a container offers the initial option for hydrogen storage. To accomplish the high-pressure storage (350 bar to 700 bar), a special tank material is required, which must be lightweight, adequate strength, good thermal conductivity, and economical. Take advantage of the relatively low price and high thermic conductivity, some steel- and aluminum-type materials are utilized in the construction of storage tanks [31, 32]. Nevertheless, these metal materials are nondurable, heavy-weight, and increase safety concerns. In contrast to the metal material, carbon fiber reinforced plastic (CFRP) [32–34], with lightweight, sufficient strength, and durable properties, serves as a more promising material for the pressurized gas vessel. While the relatively low thermal conductivity and high-price issues need to be solved before the CFRP extensively be used. Underground salt caverns can perform high-pressure gas storage and are applied to stationary store pressured hydrogen gas [35–38]. It is a feasible option for compressed hydrogen gas storage, with adjustable storage capacity, high-pressure storage ability, adaptable operating pressure, and minimized hydrogen leakage. Nevertheless, the salt cavern needs solution mining water and brine disposal, near the location of hydrogen production or consumption, and suitable geology, which are significant challenges for its development.

The hydrogen density of the compressed hydrogen gas is 24 g/L at 350 bar and 40 g/L at 700 bar (300 K) (Table 2). Because of the increased density, higher hydrogen storage capacity can be achieved (*i.e.*, 0.052 kg H_2_/kg system and 0.0277 kg/L at 700 bar, Table 2). However, they still far short of the ultimate DOE capacity goals for onboard storage for hydrogen-powered vehicles (0.065 kg H_2_/kg system and 0.050 kg/L, Table 1). Moreover, the high cost and safety concerns caused by the high-pressure operation are obstacles to the full development of the compressed gas storage system.

#### 2.1.2. Cryogenic Storage

Another way to increase the volumetric density of hydrogen is liquefaction. The density of the liquid hydrogen is 70 g/L at 1 bar and 20 K. Even though higher hydrogen storage capacity can be obtained theoretically, the practice efficiency of the liquid hydrogen tank restricts its applications. Due to the low boiling point of the liquid hydrogen (20 K), a special designed metallic double-walled container with the excellent insulation system is necessary to maintain the cryogenic temperature.

The most important concern for cryogenic storage is the hydrogen boil-off. To date, even the best-insulated container may occur hydrogen evaporation, container pressure increase, and result in energy efficiency problems as well as security problems. Also, the high energy requirements (30 to 33% of the total energy) for hydrogen liquefying and cost consideration are hindrances of its further development [33, 39].

#### 2.1.3. Cryocompressed Storage

Considering the advantage and disadvantage of the compressed gas and cryogenic storage, an alternative hydrogen storage approach, cryocompressed storage, has been developed. The relatively low pressure of this storage method reduces the demand for the expensive CFRP tank. Additionally, it can minimize hydrogen boil-off and improve energy efficiency.

In the cryopressurizing storage approach, the density of hydrogen increases to around 87 g/L at 276 bar at 20 K. The hydrogen storage capacity in the cryopressurized container increases to 0.058 kg H_2_/kg system and 0.043 kg/L, which meets the goal of DOE 2025 onboard storage values. Although the relatively higher storage capacity has been achieved, more vital improvements, including milder the storage, distribution, and operation condition, improve the hydrogen capacity, reduce the overall cost, and need to be made to realize the sustainable hydrogen economy development [40, 41].

### 2.2. Solid Hydrogen Storage Systems

**Figure 2:**
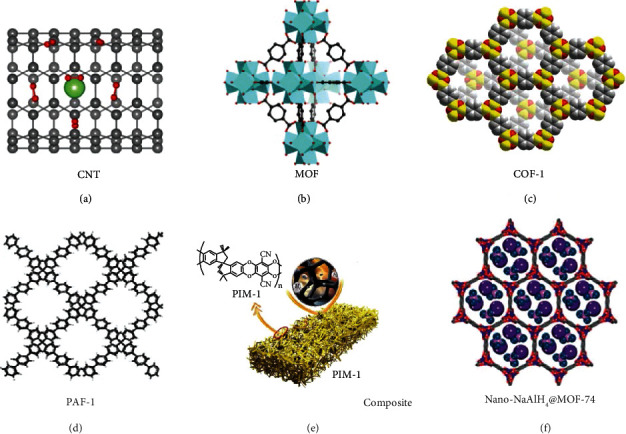
Overview and examples of the solid hydrogen storage systems. (a) Nanoporous carbon materials (carbon nanotube (CNT), (b) MOF, (c) COF, (d) PAFs, (e) the structure of a nanoporous organic polymer (PIM-1) and the composite with a nanoporous filler, and (f) nanohydrides (sodium alanate (NaAlH_4_)) confined in the nanopores of a MOF (MOF-74) [27, 51–56]. Reproduced with permission [56]. Copyright 2019, American Chemical Society. Reproduced with permission [57]. Copyright 2018, Wiley-VCH. Reproduced with permission [52]. Copyright 2015, American Chemical Society. Reproduced with permission [53]. Copyright 2009, Wiley-VCH. Reproduced with permission [53]. Copyright 2009, Wiley-VCH. Reproduced with permission [55]. Copyright 2013, Materials Research Society and Cambridge University Press.

In contrast to conventional storage approaches, material-based methods rely on physisorption and/or chemisorption to immobilize and store hydrogen in solid-state. Material-based hydrogen storage is generally considered a safer and practical alternative to conventional liquid or gaseous storage due to the stable energy states of the hydrogen composites, low operational hindrance, and release-on-demand nature [42–45]. In the solid hydrogen storage system, the interaction between the hydrogen and nanomaterials seriously affects the material hydrogen storage performance. Typical three absorption processes are summarized (Table 3) [46]. The first type is molecular hydrogen weakly bonded or trapped on the surface of nanoporous materials (adsorbents) via van der Waals interactions. However, due to the weak interaction energy (generally less than 10 kJ mol^–1^) between the matrix and the nonpolar hydrogen molecules, the immobilized hydrogen could be spontaneously released from the matrix at high temperature. Therefore, cryogenic temperature, *e.g.*, 77 K (–196°C), is commonly applied for the storage capability evaluation. On the other hand, hydrogen storage and applications at room temperature are practically desired but required great efforts to achieve [47, 48]. Porous nanostructured materials with high surface area are advantageous to increase the hydrogen storage density due to their low density and high porosity [44, 49, 50]. Nanoporous carbon materials (Section 2.2.1), metal-organic frameworks (MOFs, Section 2.2.2), covalent organic frameworks (COFs, Section 2.2.3), porous aromatic frameworks (PAFs, Section 2.2.4), and nanoporous organic polymers (Section 2.2.5) are widely investigated examples for hydrogen storage and will be introduced below (Figure 2). The second type of interaction between the hydrogen and nanomaterials is atomic hydrogen form chemical bond that strongly binds with material (the interaction energy is arranged from 100 to 200 kJ mol^–1^). In chemical storage approaches, hydrogen is stored in a solid materials by chemical bonding and released through chemical reactions under specific conditions. In this review, nanoscale hydrides will be discussed separately in the following subsection. The last hydrogen/materials interaction type is quasimolecular interaction or termed Kubas interaction. It is energetically between physisorption and chemisorption, with an enthalpy of -20 to -70 kJ/mol H_2_ and a binding energy between 0.1 and 0.8 eV. In this case, the covalent bond between the hydrogen molecules is weakened by the charge transfer or the polarization induced by the metal in the nanomaterials, leading to a shorter distance between the molecular hydrogen and material (~0.254 nm for the Kubas interaction vs. more than 0.3 nm for the physisorption). Accordingly, based on such designing principles, an ideal hydrogen storage material with high storage capacity and outstanding ab(de)sorption properties at mild condition is promising to be achieved shortly.

#### 2.2.1. Nanoporous Carbon Materials

Due to the high porosity, low density, and cost efficiency, nanoporous carbon materials have been considered to be promising carriers for hydrogen storage [58, 59]. Nanoporous carbon materials and their precursors are abundant in nature with a variety of forms, *e.g.*, activated carbon (AC), carbon nanotubes (CNTs), and carbon nanofibers (CNFs). These nanoporous carbon materials show a broad diversity in their material structures and synthetic approaches, offering varied compositions, pore sizes, surface areas, and functionalities for hydrogen storage.  Table 4 summarized the selected nanoporous carbon materials and their hydrogen storage properties.

AC has been regarded as a potential candidate for gas storage purposes due to its extremely low cost, commercial availability, and availability on chemical modification [60, 61]. AC generally displays a high degree of porosity, indicating a surface in exceed 3000 m^2^ g^–1^. Generally, the physical adsorption of hydrogen in carbon materials follows the Langmuir isotherm model, indicating a monolayer adsorption on the surface [62, 63]. The high surface area of AC enhances the physical adsorption capacity, particularly at cryogenic temperature and high pressure. However, due to the thermal instability of the absorption interaction (van der Waals force), modification of AC to increase the heat of adsorption between AC and hydrogen molecules is vital for the improvement of gas intake capability. Theoretically, the hydrogen uptake of AC could achieve 4.0 wt% at 77 K but less than 1.0 wt% at room temperature and 100 bar, leading to poor commercial practicality [64, 65]. Chemical modifications, such as potassium hydroxide (KOH) treatment and metal doping, are applied to improve the hydrogen storage performance of AC [66–68]. Sevilla, Mokaya, and Fuertes reported an AC material preparing from a polypyrrole precursor and KOH treatment exhibited a high surface area as 3000−3500 m^2^ g^–1^ and hydrogen storage capacity of up to 7.03 wt% at 77 K and 20 bar [69].

**Figure 3:**
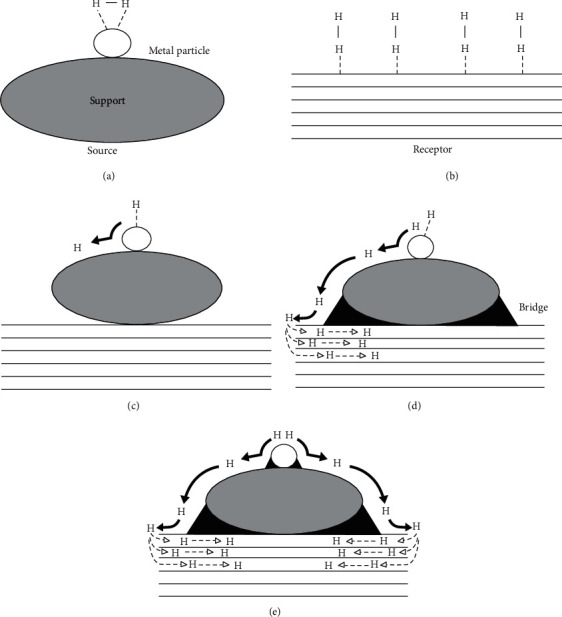
Hydrogen spillover mechanism in a supported catalyst system: (a) adsorption of hydrogen on a supported metal particle; (b) the low-capacity receptor; (c) primary spillover of atomic hydrogen to the support; (d) secondary spillover to the receptor enhanced by a physical bridge; (e) primary and secondary spillover enhancement by improved contacts and bridges. Reproduced with permission [71]. Copyright 2005, American Chemical Society.

The hydrogen spillover technique has been reported as an effective approach to enhance the binding energy between hydrogen molecules and carbon material surfaces at room temperature [70–72]. Hydrogen spillover is a multiple-step process, including dissociation of hydrogen from the metal surface, hydrogen diffusion to the support surface, and combination/desorption cycles of the mobile hydrogen species on the support surface, although investigation is still processing to discover the underlying mechanism of spillover effect (Figure 3). Doping with transitional metals, such as platinum (Pt), palladium (Pd), and nickel (Ni), on hydrogen storage materials was found to increase the hydrogen storage capability and stability due to spillover phenomenon. ACs with Pt and Pd doping demonstrated 1.10 wt% and 5.50 wt% hydrogen intake capabilities at 298 K with 100 bar and 80 bar, respectively [73–75]. Several green processes have also been applied to produce AC by using plant fibers, coconut shell, or oilseeds as the raw materials [76]. Ngadi and the coworkers reported the use of fruit bunch to produce AC for hydrogen storage, showing a maximal 2.14 wt% H_2_ intake at 77 K and 19 bar [77].

Hydrogen storage in carbon nanotubes (CNTs) has also been intensively investigated. CNTs are with diameters in the range of a nanometer and refer to single-wall carbon nanotubes (SWCNTs) and multiwall carbon nanotubes (MWCNTs) consisting of nested single-wall carbon nanotubes [78, 79]. Such nanostructure allows CNTs to store hydrogen in their microscopic pores or within the tube structures, possessing an estimated capacity of 5 to 10 wt% based on the early work of Dillon and the coworkers in 1997 [80]. Experimental results showed that the hydrogen storage capacity of SWCNTs and MWCNTs reached 4.5–8 wt% at 77 K and a moderate capacity of approximate 1 wt% at ambient temperature and pressure [81–83]. The hydrogen storage capacity of MWCNTs could be significantly improved under high-pressure environments, *e.g.*, 2.0 wt% at 40 bar, 4.0 wt% at 100 bar, and 6.3 wt% at 148 bar, at room temperature [84]. Similar with AC, mental-doping is also effective to enhance the storage capability of CNTs. It was reported that the Li-doped MWCNTs offered a hydrogen uptake up to 20 wt% at room temperature and 1 bar [85]. The other potassium- (K-) doped MWCNTs could also achieve up to 14 wt% hydrogen uptakes under ambient conditions [85]. Moshfegh *et al.* expended the scope of doping elements to calcium (Ca), cobalt (Co), iron (Fe), Ni, and Pd on MWCNTs and investigated their hydrogen storage capabilities, showing 0.3 wt%, 1.05 wt%, 1.5 wt%, 0.75 wt%, 0.4 wt%, and 7.0 wt% hydrogen intakes under ambient conditions, respectively [86]. This result indicated Pd would be the most promising doping element. It is noted that the defects, such as pentagon-heptagon pair, the substitution of heteroatoms (B, N, or P), and topological distortion, could improve the hydrogen adsorption binding energies and storage capability of SWCNTs [51, 87].

Another nanoporous carbon material for hydrogen storage application is carbon nanofibers (CNFs). CNFs exhibit a high surface area and excellent mechanical properties. Synthesis methods of CNFs include chemical vapor deposition, electrospinning technique, and templating methods [88]. These methods are simple and suitable for mass production, which make CNFs as a potential candidate due to the low cost and commercial availability. Early researches indicated that the hydrogen storage capability of CNFs ranged from 0.7 wt% to 6.54 wt% at room temperature and approximately 100 bar [89–91]. The deviation was attributed to the manufacturing methods of CNFs. In this decade, CNFs obtained by chemical activation treatment are of great interest due to their increased surface area and controllable pore sizes [92–96]. As the other carbon materials, chemical treatment by using hydroxide salts, carbonate salts, zinc chloride, and phosphoric acid was reported. Ni-doped GNFs obtained by metal doping showed an enhanced hydrogen uptake as 2.2 wt% at 298 K and 100 bar [97]. It is noted that the hydrogen storage capability of nanoporous carbon materials is highly dependent on their fabrication methods, shapes, impurity contents, oxygen-containing functionalities, and adsorbed (doping) species. The mass production of nanoporous carbon materials with stable qualities and cost efficiency is highly important for commercial applications. Finally, except AC, CNTs, and CNFs, other nanoporous carbon materials, *e.g.*, zeolite, graphene, graphite oxide, and fullerene, are also studied and used for hydrogen storage materials [58, 59, 98, 99]. The diversified structures and naturally abundance of these carbon materials are highly beneficial for material design and production to match the requirement of hydrogen storage purpose.

#### 2.2.2. Metal-Organic Framework

**Figure 4:**
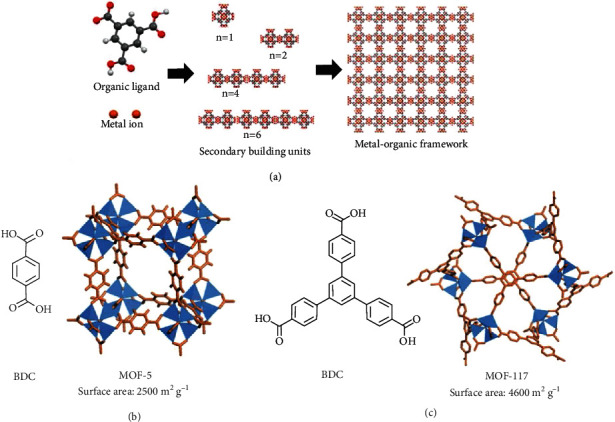
(a) Schematic illustration of the mechanism and formation of metal-organic frameworks (MOFs) [102]. (b) Chemical structure of BDC and MOF-5 and (c) BDC and MOF-177. The structures of MOFs are reproduced with permission [109]. Reproduced with permission [102]. Copyright 2019, Multidisciplinary Digital Publishing Institute. Reproduced with permission [109]. Copyright 2012, American Chemical Society.

Metal-organic frameworks (MOFs) are crystalline porous materials consisting of metal ion clusters and organic ligands (Figure 4(a)) [57, 100–107]. MOFs are highly porous with micropores (<2 nm) and a continuous skeleton. Several synthetic methods have been developed to produce two-dimensional (2D) and three-dimensional (3D) MOFs through coupling metal-containing clusters with multidentate organic ligands, *e.g.*, sulfonates, carboxylates, imidazolates, and tetrazolates [108, 109]. Thus, the selection of metal ions and organic building blocks allows the control of framework topology, pore size, and surface area. Due to the defined structures with high porosity and surface area, MOFs have been attracting extensive interest as a powerful candidate in the field of gas storage in the past two decades. Several comprehensive reviews have shown the design, synthesis, and applications of MOFs for hydrogen storage [57, 109–111]. The examples of MOFs and their hydrogen storage properties are summarized in Table 5.

The first example of using MOFs for hydrogen storage applications was reported by Yaghi's group in 2003 [112]. MOF-5 was synthesized from zinc salt and 1,4-benzenedicarboxylic acid (BDC) to give Zn_4_O(BDC)_3_ and exhibited a hydrogen intake of 4.5 wt% at 78 K and 1.0 wt% at room temperature and 20 bar, opening a new avenue for hydrogen storage materials (Figure 4(b)). However, MOF-5 exhibited poor moisture-stability, leading to limited applicable environments and unstable performance. The change in structure topology and chemical linkage resulted in enhanced moisture, thermal, mechanical, and acid/base stabilities as well as porosity. For example, the BDC linker in MOF-5 was replaced with other rigid and bulky moieties, *e.g.*, 1,3,5-benzenetribenzoate (BTB), to increase the porosity and hydrogen storage capacities (Figure 4(c)) [113, 114]. The obtained MOF-177 carried a BET surface area of 4600 m^2^ g^–1^ and a hydrogen intake of 7.5 wt% at 77 K and 70 bar. The substitution of linkers, metal ions, and functional groups was applied to synthesize a series of isoreticular MOFs for improved stability and capability [100]. For example, the introduce ethynylene units into the *p*-phenylene and the carboxylic groups in MOF-5 give novel COFs with similar skeletons but with superior properties [52, 115, 116]. The obtained NU-100 has a BET surface area of 6143 m^2^ g^–1^ and a hydrogen uptake capacity of 10.0 wt% at 77 K and 56 bar. The other NU-110 has a BET surface area of 7140 m^2^ g^–1^ with a hydrogen capacity of 8.82 wt% at 77 K and 45 bar. Another MOF-399 with a high surface area of 7157 m^2^/g also shown a high hydrogen uptake capability of 9.02 wt% at 77 K and 45 bar [116]. Several metal ions could be applied to build MOFs. Férey and coworkers reported chromium- and aluminum-based MOFs, *i.e.*, Cr-MIL-53 and Al-MIL-53, showing hydrogen uptakes of 3.1 wt% and 3.8 wt% at 77 K and 16 bar, respectively [117]. Panella *et al.* used copper(II) ions and benzene-1,3,5-tricarboxylate (BTC) to prepare a MOF (Cu-MOF-5), Cu_3_(BTC)_2_, exhibiting BET surface area of 1154 m^2^ g^–1^ and a maximum hydrogen uptake of 3.6 wt% at 77 K and 0.35 wt% at room temperature and 65 bar [118]. These results indicated the properties, and pore sizes of MOFs could be tuned by selecting the building blocks and linkers.

However, one of the major limitations of MOF is the weak van der Waals interaction between hydrogen atoms and MOFs. The isosteric heat for hydrogen adsorption of MOFs is generally less than 10 kJ mol^–1^ [109, 119]. This weak interaction energy is strong enough for applying MOFs for hydrogen storage under cryogenic conditions. However, the storage capability of MOFs sharply deteriorates when operating at room temperature. It is noted that the isosteric heat should be increase to 15–20 kJ mol^–1^ to stabilize the hydrogen atoms on MOF surfaces at room temperature [120]. One of the highest capacities of hydrogen storage in MOFs was achieved by using MOF-210 as 17.6 wt% at 77 K and 80 bar; however, the uptake decreased to 2.7 wt% at 298 K and 80 bar [28]. The introduction of active metal sites and the control of pore size and functionalization in MOFs are two well-developed strategies to improve the isosteric heat. A number of metals, *e.g*., lithium (Li), sodium (Na), K, Magnesium (Mg), Ca, beryllium (Be), titanium (Ti), Pt, Pd, copper (Cu), Fe, Co, Ni, and zinc (Zn), in element or ion forms have been applied as clusters or on MOF decoration [121]. Long and coworkers reported the use of Be_12_(OH)_12_(BTB)_4_ MOF (Be-MOF) could absorb hydrogen with 2.3 wt% at 298 K and 95 bar, which is a sharp contrast as topically similar MOF-177 with a 0.62 wt% hydrogen uptake at 298 K and 100 bar [122]. Detailed discussions on MOF modification and doping for gas storage applications could be found in other reviews [57, 109–111, 123]. The varied design and synthesis from extended organic building blocks to functional MOFs are still a popular and impactful research topic.

In addition to experimental investigations, some theoretical studies of molecular simulations have also been performed for clarifying the mechanism and screening high hydrogen capture materials. Space and the coworkers used Monte Carlo simulation to model hydrogen sorption in MOFs [104]. It demonstrated that the MOFs should have relatively small pores and interconnected pores with high surface area to create strong MOF-H_2_ interactions. Additionally, polarization interactions also played a critical role for hydrogen stabilization in MOFs. Froudakis and coworkers applied *ab initio* calculations to confirm that the interaction energies between the hydrogen molecules and the Li-modified MOFs are significantly enhanced, which can be contributed to the high degree of polarization of hydrogen molecules [105]. Therefore, MOFs with a charged or doped framework with narrow pores and exceptional internal surface area for enhancing their van der Waals interaction and polarization interaction (Kubas-type interaction) are excellent hydrogen storage candidates. The theoretical investigation of hydrogen storage in MOFs and COFs is summarized in the other review [106]. Recent advances in high-performance computers allow first-principles molecular dynamics to simulate the various diffusion processes of hydrogen molecules inside MOF structures, providing a clear picture of the diffusion mechanism of hydrogen molecules in nanoporous materials [107].

#### 2.2.3. Covalent Organic Framework

Nanoporous organic polymers consisting of organic skeletons with lightweight elements, such as C, H, N, O, and B, exhibited low density, low cost, high stability, and structure versatility [124–127]. Like MOFs, these nanoporous organic polymers have attracted great interest for hydrogen storage owing to their high surface areas. Moreover, nanoporous organic polymers present advantages over MOFs and carbon materials on their tunable structures and postsynthetic functionalization through sophisticated synthetic and polymerization techniques [128, 129].

**Figure 5:**
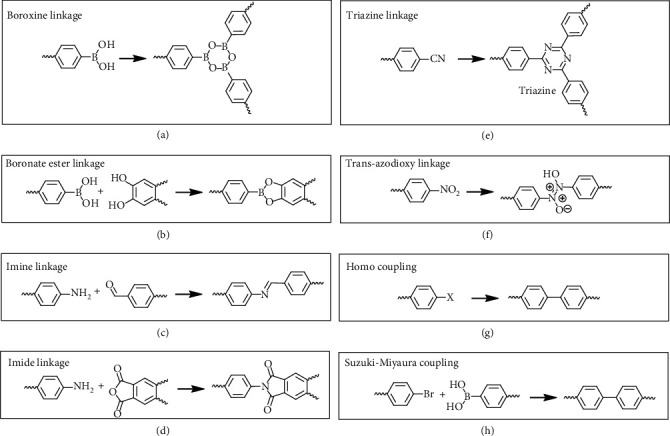
Examples of chemical reactions for synthesizing covalent organic frameworks.

COFs are carbon-based crystalline nanoporous organic polymers and constructed with strong covalent linkages, *e.g.*, B-O, C-O, B-C, C-C, and C-N, to give 2D and 3D structures.  Figure 5 shows examples of chemical reactions for synthesizing COFs. COFs are with high porosity, well-order pores, and superior chemical and thermal stability [53, 130]. Due to the above advantages, COFs as one of the powerful candidates for hydrogen storage have been researched theoretically and experimentally in the past 15 years [131, 132]. The examples of COFs and their applications on hydrogen storage are shown in Table 5. The design, synthesis, and applications beyond hydrogen storage of COFs have been summarized in other reviews [124–127].

**Figure 6:**
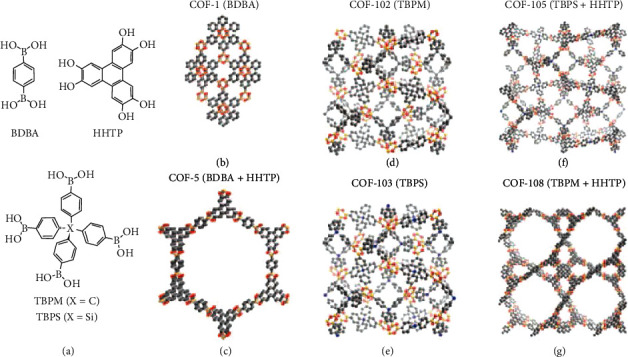
Molecular structures of building units (a) and crystal structures of COFs (b–g). Hydrogen atoms are omitted for clarity. Carbon, boron, oxygen, and silicon atoms are represented as gray, orange, red, and blue spheres, respectively. Reproduced with permission [135]. Copyright 2008, American Chemical Society.

In 2005, Yaghi's group pioneered the research on COFs and reported a series of porous COFs [133]. For instance, COF-1 was synthesized by using a self-condensation reaction of 1,4-benzenediboronic acid, and COF-5 was prepared from 1,4-benzenediboronic acid and 2,3,6,7,10,11-hexahydroxytriphenylene. COF-5 exhibited a high BET surface area as 1590 m^2^ g^–1^ and a 3.5 wt% hydrogen intake at 77 K and 80 bar. Another 3D-COFs family, including COF-102, COF-105, and COF-108, were also synthesized, showing a larger surface area than 2D COF-5 [134]. Among these COFs, COF-102 has a BET surface area of 3620 m^2^ g^–1^, and its hydrogen uptake capacities are 7.2 wt% at 77 K and 35 bar and 10.0 wt% at 77 K and 100 bar (Figure 6) [135–138]. It is also noted that the hydrogen volumetric uptake of COF-102 achieved 40.4 g L^–1^, which is the best performance of these 3D-COFs. The phenylene groups in the COF-102 backbone could be substituted by diphenyl, triphenyl, and naphthalene pyrene groups, giving COF-102-2, COF-102-3, COF-102-4, and COF-102-5, respectively [139]. The modulation of the backbone and pore size could further control the hydrogen uptake capacity of COFs. COF-102-3 demonstrated 26.7 wt% and 6.5 wt% hydrogen uptakes at 77 and 300 K under 100 bar, respectively. The hydrogen uptakes of COF-105 and COF-108 at 77 K are 10.0 wt% at 80 bar for COF-105, and 10.0 wt% at 100 bar for COF-108. Zheng and coworkers reported a bowl-shaped COF from cyclotricatechylene (CTC) and 1,4-benzenediboronic acid [140]. The CTC-COF has a BET surface area of 1710 m^2^ g^–1^ and a hydrogen uptake of 1.12 wt% at 77 K and 1.1 bar. The superior hydrogen uptake capacity of CTC-COF could be attributed to the additional adsorption in the bowl-shaped CTC cavity.

COFs present excellent hydrogen storage capacity under high pressure. However, as MOFs, the capacity deteriorates sharply when operating at an increased temperature. To overcome this problem, metal-doping has been applied to COFs to improve the hydrogen storage capacity [129, 132]. Several computational and experimental studies have been reported to use metal ions or elements, such as Li, Mg, Ti, and Pd, for COF doping and the improvement of hydrogen uptake capabilities under practical conditions [141–144]. The metal decoration on COF skeletons enhances the interactions between the COF skeleton and hydrogen atoms. It was reported that the lithium doped COF-105 and COF-108 exhibited an improved hydrogen storage capacity of 6.84 wt% and 6.73 wt% at 298 K and 100 bar [145]. Such enhancement of hydrogen uptakes is because of the generation of the dative bonds between positively charged lithium ions and hydrogen molecules. Pramudya and Mendoza-Cortes systematically studied the effect of doping elements on several COFs with imine and hydrazide linkage [146]. Such linkers in the COFs could chelate with Co(II), Cu(II), Fe(II), Mn(II), and Ni(II) anions. It was determined that Co-, Ni-, and Fe-doping effective enhanced the hydrogen uptake capabilities of COFs at 298 K. The synthesized COF-340 with Co(II)-doping exhibited the highest hydrogen uptake of 7.00 wt% at 298 K and 250 bar.

#### 2.2.4. Porous Aromatic Framework

Porous aromatic frameworks (PAFs) are one of the porous organic materials with a tetrahedrally diamond-like structure [147, 148]. PAFs exhibit similar properties to those of COFs, such as high porosity, large surface area, low mass densities, and high thermal and mechanical stability. Additionally, the organic frameworks facilitate the postsynthetic functionalization and pore size modulation, further improving the capabilities. PAFs contain multiple phenyl rings and are generally synthesized via irreversible cross-coupling reactions, which is different from the preparation of COFs by using reversible condensation reactions. Due to unique structure and properties, PAFs have found several applications in hydrogen storage, molecular separation, catalysis, and molecule sensing.

**Figure 7:**
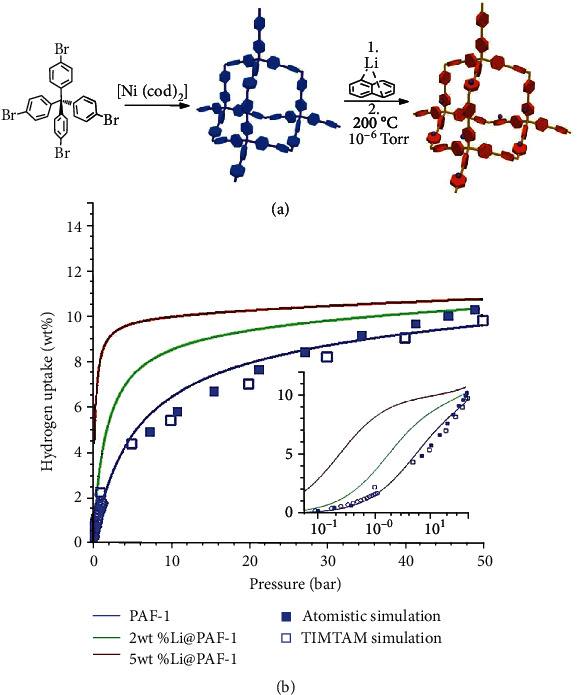
(a) Schematic illustration of the synthesis of PAF-1 and Li-doped PAF-1. (b) Computed hydrogen total uptake at 77 K. The inset (b) shows the logarithmic graph of hydrogen total uptake at 77 K. Reproduced with permission [153]. Copyright 2012, Wiley-VCH.

In 2009, Zhu and the coworkers reported the first synthesis of PAFs [27]. The PAF was termed PAF-1 and exhibits a BET surface area of 5600 m^2^ g^–1^. Such a high surface of PAF-1 allows high uptakes of hydrogen (7.0 wt% at 77 K and 48 bar). However, PAF-1 shows relatively low heat of adsorption as 4.6 kJ mol^–1^, suggesting a weak interaction between hydrogen molecules and the surface of PAF-1. This result suggests the hydrogen intake capacity of PAF-1 becomes poor at increased temperature or under an ambient pressure. Therefore, a series of PAFs with a replaced quadrivalent atom (silicon (Si) or germanium (Ge)) in lieu of the carbon center were synthesized [149, 150]. PAF-3 with Si centers exhibits BET surface area of 2932 m^2^ g^–1^, 6.6 kJ mol^–1^ heat of adsorption, and a hydrogen uptake of 5.5 wt% at 77 K and 60 bar. PAF-4 with Ge centers exhibits BET surface area of 2246 m^2^ g^–1^, 6.3 kJ mol^–1^ heat of adsorption, and a hydrogen uptake of 4.2 wt% at 77 K and 60 bar. To further improve the performance, postsynthetic modification and doping were applied to PAFs. PAF-1 could be activated by potassium hydroxide followed by a carbonized process to increase its hydrogen gravimetric capacity to 3.06 wt% at ambient pressure and 77 K [151]. Moreover, it was also reported that PAF-4 carrying lithium tetrazolide doping enhanced their hydrogen storage capacities to 20.7 wt% (at 77 K and 100 bar) and 4.9 wt% (at 233 K and 100 bar), respectively [152]. The increment of capacity could be attributed to the elevation of hydrogen binding energy in PAFs on the Li-doping sites (Figure 7) [153]. Apart from lithium, Mg- and Ca-doping were also successfully applied to increase the hydrogen binding strength of PAF, giving hydrogen storage capacities as 6.8 wt% for PAF-Mg and 6.4 wt% for PAF-Ca at 233 K and 100 bar [154]. The other series of PAFs were constructed by diphenylacetylene derivatives as the linkers. The synthesized PAF-324 and PAF-334 were reported to have significantly high hydrogen uptakes of 6.32 wt% and 16.03 wt% at 298 K and 100 bar, respectively [155]. These hydrogen storage properties of PAFs are summarized in Table 5.

In comparison with MOFs and COFs, only limited PAFs have been developed and the hydrogen storage capacity still needs further improvement to match practical demands. Through the use of suitable building units and doping materials, it is possible to increase the volumetric capacities and heat of adsorption. A detailed review of the synthesis and applications of PAFs can be found elsewhere [147].

#### 2.2.5. Nanoporous Organic Polymers

**Figure 8:**
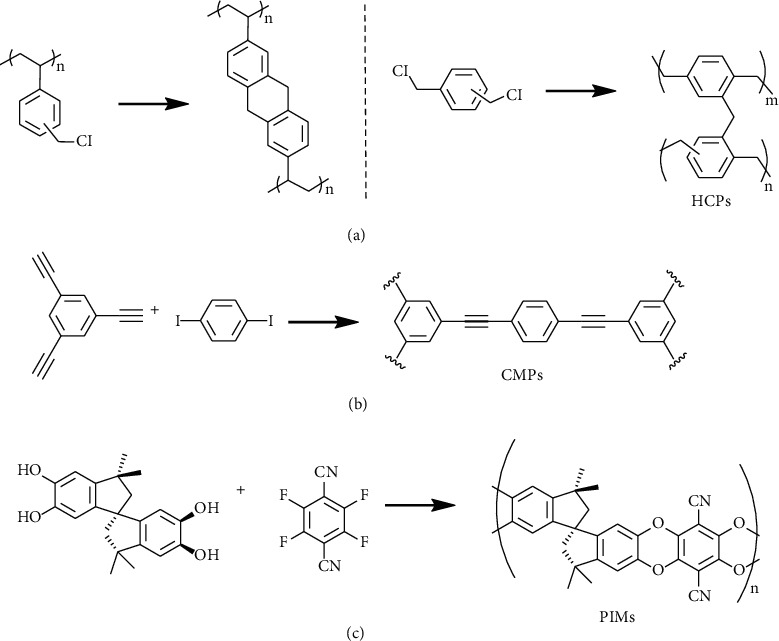
Synthetic routes of (a) hypercrosslinked polymers (HCPs), (b) conjugated microporous polymers (CMPs), and (c) polymers of intrinsic microporosity (PIMs).

Although COFs and PAFs provide promising properties on the surface area and capability, COFs and PAFs are generally not cost-effective and offer powder-type polymers with relatively poor processability and mechanical properties, limiting their practicality. Other nanoporous organic polymers, such as hypercrosslinked polymers (HCPs), conjugated microporous polymers (CMPs), and polymers of intrinsic microporosity (PIMs), have also been widely used as adsorbents, separation materials, catalyst carriers, and gas storage materials [156–158]. HCPs, CMPs, and PIMs possess several advantages and unique properties for hydrogen storage applications, including (1) variable polymer backbones and facile functionalization, (2) tunable pose size and crosslinking density, (3) light-weight and high surface area, (4) high processability for bulk, coating, and composite materials, and (5) low-cost and accessibility for mass-production.  Figure 8 demonstrates structural examples of HCPs, CMPs, and PIMs. However, in comparison with COFs and PAFs, these nanoporous organic polymers exhibit relatively low surface area (<2000 m^2^ g^–1^), which limits the hydrogen uptakes [159, 160]. The hydrogen storage properties of selected HCPs, CMPs, and PIMs are summarized in Table 6.

HCPs are amorphous polymers was the first synthesis in the 1940s and found broad applications for column chromatography applications in the 1970s [161]. A variety of organic synthesis techniques were applied to give HCPs, allowing HCPs with controllable pore sizes and surface areas [162, 163]. The surface area of HCP could theoretically achieve more than 2000 m^2^ g^–1^. The early studies of HCPs for hydrogen storage are reported in 2006. Svec *et al.* synthesized polystyrene-type HCPs with BET surface areas of 1930 m^2^ g^–1^ and high uptakes of hydrogen as 1.5 wt% at 77 K and 1.2 bar [29]. Cooper and the coworkers also utilized the other polystyrene-type HCPs with BET surface areas of 2090 m^2^ g^–1^ for hydrogen storage, showing hydrogen uptakes of 3.04 wt% at 77 K and 15 bar [164]. The other research reported by Cooper's group was a polyphenylene-type HCP synthesized from *para*-dichloroxylene and 4,4′-bis(chloromethyl)-1,1′-biphenyl. This HCP exhibited the surface area of 1904 m^2^ g^–1^ and a hydrogen uptake of 3.68 wt% at 77 K and 15 bar [165]. Germain, Svec, and Fréchet further synthesized polyaminobenzene HCP with aromatic ring backbones via Buchwald coupling of *p*-diaminobenzene and tribromobenzene [166]. The HCP showed an extremely high isosteric heat as 18 kJ mol^–1^ of hydrogen adsorption due to its small pores (<0.7 nm) although a low experimental hydrogen intake of 0.22 wt% at 273 K and 9 MPa was obtained. Liang, Tan, and coworkers reported a polyphenylene-type HCP and Pt nanoparticle composite [167]. The spillover effect of Pt nanoparticle improved the hydrogen capability, showing a 0.21 wt% hydrogen intake at 273 K and 9 MPa.

CMPs are another type of nanoporous organic polymers carrying multiple carbon-carbon triple bonds and/or aromatic rings to form *π*-conjugated skeletons [168, 169]. The first hydrogen storage application of CMP, *i.e.*, a poly(aryleneethynylene) network, was developed by Cooper group in 2007 [170]. The poly(aryleneethynylene) CMPs obtained by Sonogashira coupling reaction exhibited a large BET surface area of 1018 m^2^ g^–1^ and a hydrogen uptake of 1.4 wt% at 77 K and 1.0 bar. After that, various CMPs with diverse structures and porosity were developed. Specific surface areas as high as 1200 m^2^ g^–1^ have been achieved by using sterically demanding linkers with a trigonal or tetragonal geometry [171, 172]. Alkyne linkers could be polymerized by using Yamamoto reaction or Ni-catalyzed Ullmann coupling reaction gave CMPs with specific surface areas up to 842 m^2^ g^–1^ and a hydrogen intake of 131 cm^3^ g^–1^ at 77 K and 1.13 bar [173]. Similar network topologies with comparable pore properties and specific surface areas of up to 1380 m^2^ g^–1^ were synthesized by using Pd-catalyzed Suzuki–Miyaura coupling of aromatic halides with boronic acids. The CMP, EOF-6, exhibited a hydrogen storage capability of 1.29 wt% at 77 K and 1 bar [174]. Several examples of nitrogen-rich CMPs were also synthesized for hydrogen storage applications [175, 176]. A CMP, PCZN-8, with a 20 mol% pyridine moiety in the backbone exhibited a BET surface area of 1126 m^2^ g^–1^ and 1.35 wt% hydrogen storage capability at 77 K and 1 bar. As nanoporous carbon materials and COFs, metal doping is an effective approach to improve the hydrogen storage of CMPs. It has been reported that CMPs with Li doping could improve the hydrogen uptake capability. Deng *et al.* demonstrated that the Li-doped CMP synthesized by homocoupling of 1,3,5-triethynylbenzene could enhance its hydrogen uptake value from 1.6 wt% for the nondoped CMP to 6.1 wt% at 77 K and 1 bar [177]. Recently, Chang *et al.* reported a novel cation–*π* induced Li-doped poly(triazatruxene) (PTAT) CMP for gas storage applications. It was found that the CMP showed a hydrogen uptake capability of 7.3 wt% at 77 K and 1 bar in comparison with that of undoped CMP with 1.9 wt% under the same conditions [178].

**Figure 9:**
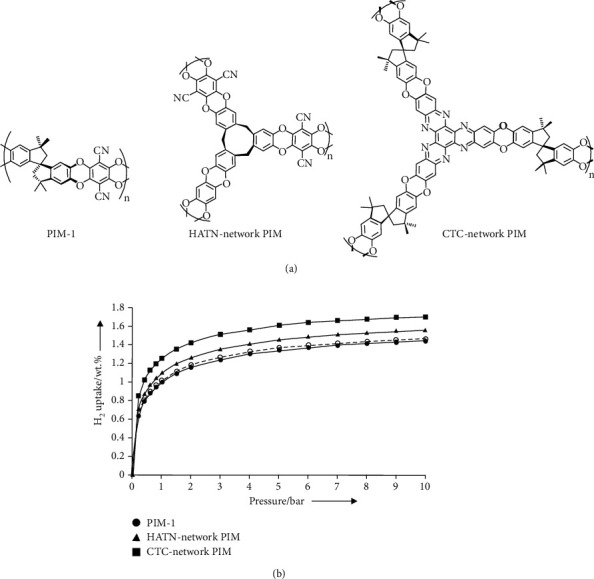
(a) Structure of PIM-1, HATN-network-PIM, and CTC-network-PIM. (b) The gravimetric hydrogen adsorption (filled symbols) and desorption (open symbols) at 77 K [181]. Copyright 2006, Wiley-VCH.

**Figure 10:**
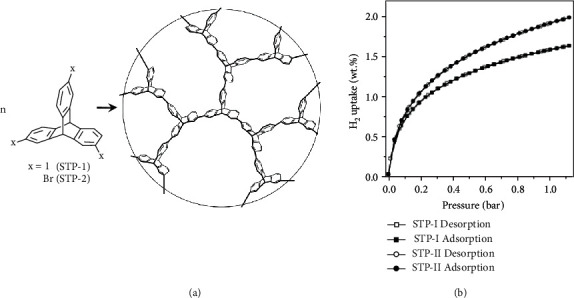
(a) Synthesis of triptycene-based porous polymers. (b) Gravimetric hydrogen adsorption and desorption profile isotherms up to 1.13 bar at 77.3 K. Reproduced with permission [184]. Copyright 2012, American Chemical Society.

PIMs are amorphous organic microporous materials with intrinsic micropores due to the connection of a bulky and planar backbone with rigid and fused ring spacers (such as a spiro-center) [179, 180]. The skeleton distortion possesses high internal molecular free volume and intrinsic microporosity. PIMs are featured with microporous materials with interconnected pores less than 2 nm diameter. Various monomers with different functional groups and torsional strain have been used to produce PIMs with desired properties such as pore size, capacity, and solubility. Early researches of using PIMs for hydrogen storage were reported by McKeown, Budd, and the coworkers, showing a cyclotricatechylene- (CTC-) based PIM-1 with a BET surface area of 760 m^2^ g^–1^ and a hydrogen uptake as 1.44 wt% at 77 K and 10 bar ((Figure 9) [181]. Thermal treatment (annealing) was also successfully applied to increase the hydrogen capability of PIM-1 to nearly double [182]. A further development of triptycene-based PIMs demonstrated an improved BET surface area up to 1990 m^2^ g^–1^ and a hydrogen uptake as 1.9 wt% at 77 K and 1.0 bar (Figure 10) [183, 184]. Recently, Webb *et al.* synthesized a novel hexaazatrinaphthylene- (HATN-) based PIMs. The HATN-PIM exhibited a surface area at 772 m^2^/g and a hydrogen intake of 3.86 wt% at 77 K and nearly120 bar [185]. The composites of PIMs with other porous polymers, such as PAFs and polyaniline, were also prepared for hydrogen storage [186, 187]. PIMs are with high processability, whereas PAFs are with high surface but with poor processability. The mixture of PIMs and PAFs provided good processability for film casting and an improved hydrogen intake capability from 2.6 wt% (pure PIM film) to 4.1 wt% (PIM/PAF (=77.5/22.5 (wt%/wt%)) film) [186]. Mays *et al.* also reported the used PIM/AC and PIM/MOF composites to prepare porous polymer-based composite membranes for mobile hydrogen storage applications [54]. The results showed that the PIM with 60 wt% AC or 40 wt% MOF could be used for film casting processes and the obtained films exhibited 1.6–2.5 times larger hydrogen intake capabilities. Such polymer/polymer and polymer/inorganic material composites offer advantages over powders in terms of safety, handling, and practical manufacturing for high-pressure hydrogen storage materials.

#### 2.2.6. Nanoscale Hydrides

In contrast to physical storage described above, metal and chemical hydrides are the other materials for hydrogen storage via chemical (ionic or covalent) bonding [188–191]. These hydrides generally consist of a metal cation and an anion with hydrogen. Hydrides are promising materials for storage applications due to the high hydrogen densities and relatively high safety (low reactivity). The use of light metals, *e.g.*, lithium, magnesium, and aluminum, to form hydrides offers higher gravimetric and volumetric hydrogen densities in comparison with hydrogen gas or liquid hydrogen, attracting promising applications [46]. Three types of hydrides are mainly studied for hydrogen storage [192]. The first type is metal hydrides MH_x_ (*M* is the main group or transition metal, such as Li, Na, Mg, Ca, and Ti, and *X* is the number of hydrogen atom). Hydrogen reacts with a metal or a metal alloy (M) and transfers to negatively charged hydride ions (H^−^) to generate a metal hydride, as shown in equation (1).

(1) $$\begin{array}{c} M+\left(\frac{\text{X}}{2}\right){\text{H}}_{2}\;{M\text{H}}_{x}. \end{array}$$

The second type is intermetallic hydrides, AB_x_H_y_, where *A* is typically the hydriding metal and *B* is the nonhydriding metal. The other type is termed complex hydrides, also known as chemical hydrides (MEH_x_). A chemical hydride contains a metal cation (M) and a hydrogen-containing polyatomic anion (EH_x_). Examples of EH_x_ are alanates (AlH_4_^−^), borohydrides (BH_4_^−^), and amides (NH_2_^−^).

Many metals could incorporate with hydrogen atoms to form metal hydrides. The researches of metal hydrides for hydrogen storage have been extensively studied since the 1960s [193]. Nevertheless, the main challenges using metal hydrides are still on the selection and design of metals to meet the thermodynamic and kinetic requirements of practical cyclic hydrogen insertion/removal processes. For example, aluminum hydride (AlH_3_) has the high gravimetric (10 wt% H_2_) and volumetric (148 kg H_2_ m^–3^) densities. However, the weak bonding energy of AlH_3_ (dissociation energy = −11.4 ± 0.8 kJ mol^–1^) leads to the thermodynamic instability and lack of practicality to transform Al metal to AlH_3_ under moderate conditions [194]. The drawback restricts the direct use of AlH_3_ as a hydrogen storage material. The studies on alanates and nanoscaled AlH_3_ are major alternatives of aluminum hydride. On the other hand, lithium hydride (LiH) also has a high content of hydrogen as 12.7 wt%. However, due to its highly ionic and strong bonding between Li and H, LiH generally requires a high temperature (nearly 900°C) and 1 bar for dehydrogenation, which limited further practical applications [195].

Among the light-metal hydrides, magnesium hydride (MgH_2_) has been considered as the most promising metal hydride material for hydrogen storage [196, 197]. The advantages of MgH_2_ include its high gravimetric (7.6 wt% H_2_) and volumetric (110 kg H_2_ m^–3^) densities, natural abundance, low cost, lightweight, and chemical stability. Different from AlH_3_, the main hindrance on the practicality of MgH_2_ is its high thermodynamic stability of Mg–H bonding (ΔH = 75 kJ mol^−1^ H_2_), resulting in the difficulty on hydrogen releasing. A temperature above 573 K (300°C) is essential for MgH_2_ application to accelerate hydrogen sorption/desorption under normal pressure [198]. To overcome the limitation on operating temperature, many researches have focused on the use of other metals of metal oxides as catalysts to improve sorption kinetics of Mg and reduce the activation energies of hydrogenation/dehydrogenation processes [198]. A variety of elements, *e.g.*, vanadium (V), Ti, Ni, niobium (Nb), chromium (Cr), and Fe, have been applied to prepare doped Mg through ball milling, melting, casting, and chemical processes to enhance the hydrogen storage/releasing performance [189, 192].

Chemical hydrides have attracted great interest due to their low molar mass and high gravimetric hydrogen capacity. For example, the hydrogen content of ammonia borane (NH_3_BH_3_, AB), LiBH_4_, and NaBH_4_ are 19.6 wt%, 18.6 wt%, and 10.6 wt%, respectively. Among these, AB is the most widely studied chemical hydride, with high hydrogen capacity and moderate desorption temperature, which exhibits great potential for onboard storage [26, 199–202]. It bears protic (N-H) and hydridic (B-H) hydrogens. Those two hydrogen types show an opposite polarity, which can be simply regarded as dihydrogen bonding (DHB): N-H^*δ*+^···H^*δ*-^-B, facilitating hydrogen release under relatively mild temperature (~120°C) with ultrahigh purity. The parent compound AB was first reported in 1955. AB has been received increasing efforts to investigate its thermolytic and hydrolytic dehydrogenations, due to its exceptional properties for chemical hydrogen storage (nonflammable and stable under standard conditions). AB can be synthesized through a reaction between NH_3_ and BH_3_, in which two main pathways with derived and/or modified procedures were generated. The first main pathway is a metathesis reaction in the suspension of an ammonium salt [NH_4_^+^]_x_[X^X-^] and an alkaline borohydride (LiBH_4_ or NaBH_4_) in the organic solvent at 25-45°C. The resulted H_3_NBH_3_ is not stable at ambient condition because of its instantaneous dehydrogenation. The second main pathway is an S_N_2 reaction, in which the strong base NH_3_ replaces the weak ligand L of L·BH_3_ in the organic solvent at low temperature (*e.g.*, 0°C). The reaction temperature would be raised to a higher temperature though replacement ligand L (*e.g.*, (CH_3_)_2_O or (CH_2_)_4_O) in precursors L·BH_3_ with new ligand L′ (*e.g.*, C_6_H_5_N(C_2_H_5_)_2_ or C_6_H_5_N(C_4_H_8_O)). However, the exothermicity of thermolytic and hydrolytic dehydrogenations for H_3_NBH_3_ required a chemical recycling of the related by-products. Thus, alternative pathways were developed to regenerate H_3_NBH_3_ with the by-products. The by-products of hydrolytic dehydrogenation are boric acid B(OH)_3_, tetrahydroxyborate anion B(OH)_4_^−^, and ammonium NH_4_^+^. Currently, most of regeneration focused on the formation of sodium borohydride or direct formation of H_3_NBH_3_. For the thermolytic dehydrogenation, the by-product is a mixture of polymeric residues from H_3_NBH_3_, including *trans*-*cisoid* polyaminoborane, polyiminoborane, *o*-polyborazylene, and a graphitic cross-linked polymer [203]. A lot of efforts have been made to rehydrogenate the polymer residues through the stepwise process or the one pot-process.

Despite the attractive advantages of hydrides as the hydrogen storage media, the chemical binding in the hydride is so strong that results in sluggish kinetics and unacceptable hydrogen desorption temperature, reduce their efficiency and suitability in mobile applications. The dehydrogenation of metal hydrides, such as magnesium and magnesium-based alloys hydrides, ranges from 300 to 400°C, for example. The hydrogen release temperature for chemical hydrides is relatively low but still away from the practical applications' requirement. For instance, the hydrogen desorption temperature of pure AB is around 120°C, higher than the fuel cell operation temperature (about 85°C).

**Figure 11:**
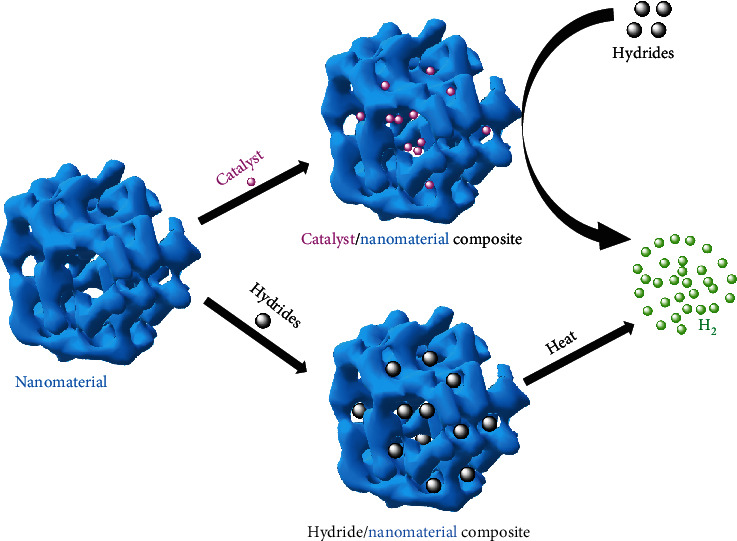
The preparation of catalyst/nanomaterial composite and hydride/nanomaterial composite.

**Figure 12:**
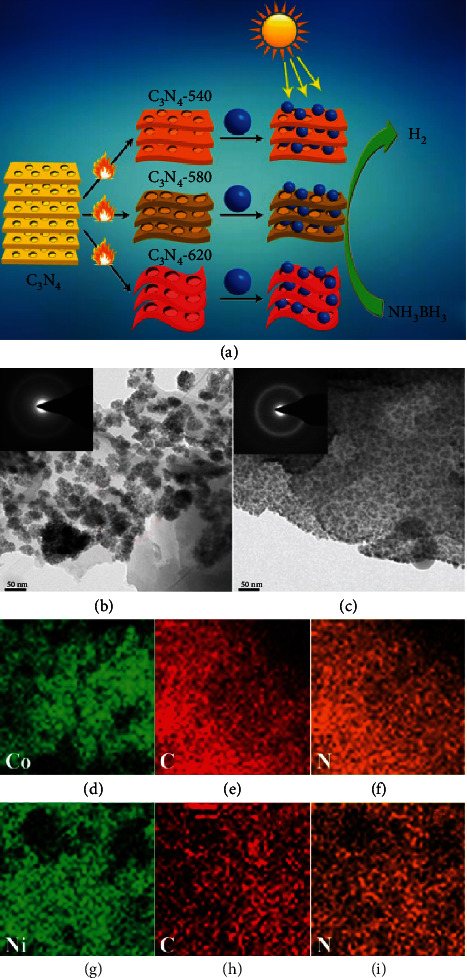
(a) The scheme diagram of the visible-light-driven catalystic procedure over based on the C_3_N_4_ with different microstructures. TEM images and the SAED patterns (insets) of (b) Co/C_3_N_4_-580 and (c) Ni/C_3_N_4_-580 and the elemental maps of Co/C_3_N_4_-580 for (d) Co, (e) C, and (f) N and Ni/C_3_N_4_-580 for (g) Ni, (h) C, and (i) N. Reproduced with permission [204]. Copyright 2017, American Chemical Society.

To further improve the hydrogen storage performance, nanotechnology is applied to combine with hydrides to either modify the catalysts or prepare nanoscale hydrides (Figure 11). A series of nonprecious C_3_N_4_ species were postmodified by thermal modification method, which were then employed to fabricate Co/C_3_N_4_ nanocomposites (NPs) with different microstructures (Figure 12) [204]. Co/C_3_N_4_ NPs were investigated in the catalytically photochemical dehydrogenation of AB at room temperature. The systematic study indicated that Co/C_3_N_4_ NPs display different catalytic activities under light irradiation. The maximum catalytic efficiency was achieved with TOF of 93.8 min^−1^ at 25°C, which is the best TOF value achieved by noble metal-free catalysts among all the reported studies. In compared with pristine C_3_N_4_, the thermally modified C_3_N_4_ species with porous structure exhibited different band structures, photoluminescence lifetime, and photocurrent density under visible light irradiation, resulting the different separation efficiency of photoinduced charge carriers. Its enlarged surface areas promoted the light absorption and separation efficiency of electrons/holes, which enhanced the efficiency of electron transfer under visible light irradiation to increase the electron density of Co NPs, leading improved photocatalytic H_2_ generation activity of that Co/C_3_N_4_ NPs. Bimetallic nonnoble CoNi NPs were successfully supported on MWNTs [205]. The ultrafine CoNi NPs with particle size of 2 nm could be monodispersed on surface of MWNTs. The synergistic effect between Co and Ni acted an important role in improvement for AB hydrolysis. The support effect could also efficiently improve the catalytic performance. Compared to the other support materials, Co_0.7_Ni_0.3_/MWNTs showed excellent catalytic performance with TOF of 128 mol_H2_ mol_cat_^−1^ min^−1^ and *E*_*a*_ as 52.1 kJ∙mol^−1^ at 45°C. Co_0.7_Ni_0.3_/MWNTs also exhibited high durability in AB hydrolysis.

Other lightweight inorganic hydrides, such as ammonia (NH_3_), hydrazine (NH_2_NH_2_), hydrazine borane (H_3_BNH_2_NH_2_), hydrazine bis(borane) (H_3_BNH_2_NH_2_BH_3_), borohydrides (BH_4_), borohydride ammoniates (M(BH_4_)_x_·(NH_3_)_y_), and alanates (AlH_4_), have displayed potential values for chemical hydrogen storage as well [206]. In order to improve the reaction rate and H_2_ yield in the thermal decomposition/hydrolysis of lightweight inorganic hydrides, a series of heterogeneous catalysts and approaches have been developed in the past few decades. Normally, heterogeneous catalysts are composited by supported monometallic, multimetallic or core-shell M(0) nanoparticles (NPs), in which NPs are deposited on the support materials, such as metal oxides, MOFs, graphene, and CNTs [207].

Cu@CoNi/graphene composites with different compositions were fabricated from trimetallic core–shell Cu@CoNi NPs and graphene in a one-step in situ reduction by using methylamine borane (CH_3_H_2_NBH_3_, MeAB, 18.0 wt% H_2_), AB or NaBH_4_, respectively. The prepared Cu-core/CoNi-shell NPs could be well dispersed on the surface of graphene [208]. Among those composites, Cu_0.1_@Co_0.45_Ni_0.45_/graphene reduced by MeAB exhibited higher catalytic activity for MeAB dehydrogenation than that reduced by AB or NaBH_4_, which displayed the best catalytic performance in the hydrolysis of MeAB with TOF of 9.4 mol_H2_∙(mol_M_)^−1^∙min^−1^ at 25°C (*E*_*a*_ = 50.7 kJ∙mol^−1^). This catalyst displayed higher catalytic activity than those of most reported noble-free-metal-based NPs, and also good durability and magnetic recyclability for the MeAB dehydrogenation. A series of M(0) NPs supported on silica, such as Fe/SiO_2_, Ru/SiO_2_, Co/SiO_2_, Rh/SiO_2_, Ir/SiO_2_, Ni/SiO_2_, Pd/SiO_2_, and Pt/SiO_2_, have been prepared for catalytic decomposition of hydrazine (NH_2_NH_2_, 12.6 wt% H_2_) to produce H_2_ [209]. All the catalysts could catalyze the selective decomposition of NH_2_NH_2_ to form H_2_ and N_2_ at temperatures higher than 300°C. Ni/SiO_2_, Pd/SiO_2_, and Pt/SiO_2_ catalysts could produce H_2_ with high selectivity under mild conditions. Among those catalysts, Ni/SiO_2_ showed the highest catalytic activity with TOF of 24 mol_H2_∙(mol_M_)^−1^∙min^−1^ and excellent H_2_ selectivity (>90%) in the NH_2_NH_2_ decomposition at 30°C. In addition, the selectivity of catalyst was temperature sensitive; the lower temperature (30-60°C) promoted the reaction to produce H_2_ over NH_3_. The poly(N-vinyl-2-pyrrolidone)- (PVP-) stabilized nickel(0) nanoparticles with an average particle size of 3.0 ± 0.7 nm could be fabricated in situ by reduction of nickel(II) 2-ethylhexanoate with hydrazine borane (H_3_BNH_2_NH_2_, HB, hydrogen capacity = 15.4 wt%) in the presence of PVP at room temperature, which were studied for catalytic methanolysis of HB [210]. Ni/PVP NPs displayed highly active and long lived in the methanolysis of HB at ambient temperature. The kinetic study revealed that Ni/PVP NPs catalyzed methanolysis is first order with regarding to catalyst concentration and zero order to substrate concentration. Ni/PVP NPs provided an initial TOF of 35.6 min^−1^ with *E*_*a*_ as 63 kJ∙mol^−1^ in hydrogen generation from the methanolysis of HB. PVP-stabilized cobalt(0) nanoclusters were prepared from the reduction of cobalt(II) chloride in the presence of PVP stabilizer in methanol [211]. Co/PVP NPs were stable in solution and could be separated as solid materials for characterization and application. Co/PVP NPs were employed in catalytic hydrolysis of NaBH_4_ (10.7 wt% H_2_) at room temperature for the portable fuel cell applications. Kinetic studies indicated that the catalytic hydrolyses of NaBH_4_ is first order regarding both of Co/PVP NPs and NaBH_4_ concentration in an aqueous medium. The Co/PVP NPs provided a lower *E*_*a*_ for the hydrolysis of NaBH_4_ both in aqueous medium (63 kJ∙mol^−1^) and in basic solution (2 wt% NaOH, 37 kJ∙mol^−1^), when compared to the reported value for bulk cobalt (75 kJ∙mol^−1^).

**Figure 13:**
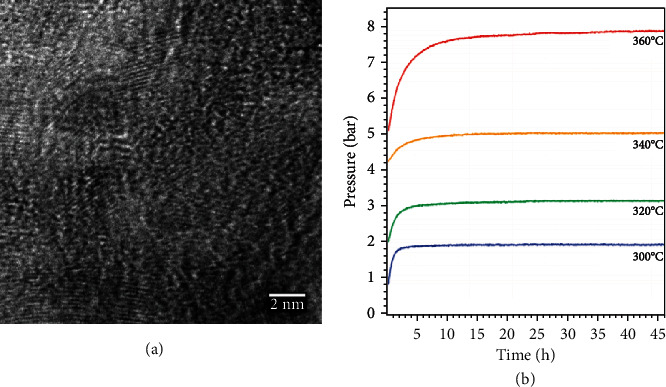
(a) TEM image of lattice fringing in MgH_2_-D occurring from the MgH_2_*hkl* = 020 plane. (b) Kinetic hydrogen desorption data for MgH_2_-D illustrating that equilibrium was reached at different temperatures. Reproduced with permission [216]. Copyright 2010, American Chemical Society.

**Figure 14:**
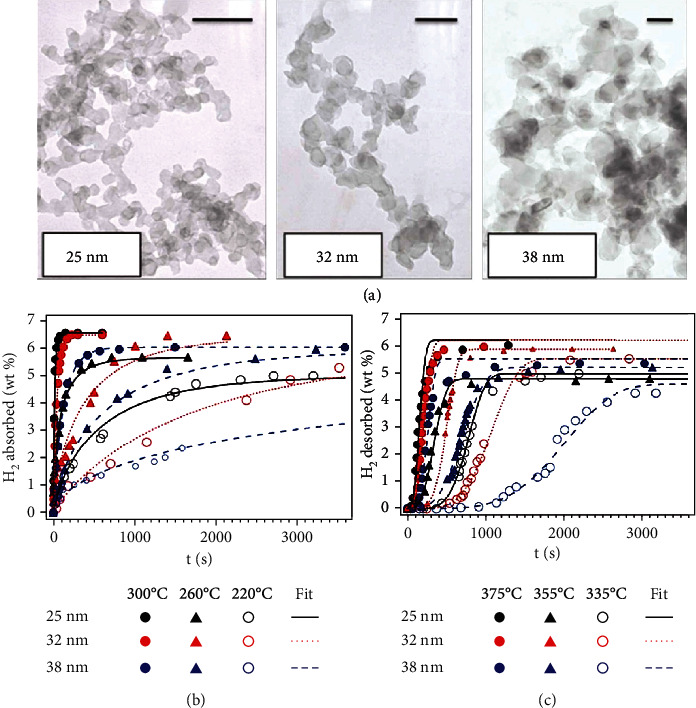
(a) TEM images of Mg nanocrystals (scale bar = 100 nm). Hydrogen (b) absorption and (c) desorption of the Mg nanocrystals at different temperatures. Reproduced with permission [220]. Copyright 2011, American Chemical Society.

Nanoscale hydrides can be prepared in two ways. One way is to direct synthesize nanostructured hydrides through physical or chemical method. The other one is via confinement of hydrides into a supporting nanomaterial. The nanostructuring hydride exhibits novel nanoarchitecture to positively change its hydrogen ab(de)sorption properties. Nanostructured magnesium hydrides with varied sizes and morphologies were extensively studied as a promising solution for hydride material practicality [192, 212–214]. Selected nanoscale metal hydrides and their properties are summarized in Table 7. Theoretically, nanostructuring metal hydrides exhibit a high active interface area and relative short hydrogen diffusion distance, which can effectively increase the storing high hydrogen content with fast kinetics. Moreover, the exposure of atoms on the nanomaterial hydride surface would weaken the Mg–H bonding due to the higher surface energy, which facilitates the hydrogen releasing. Several approaches have been applied to synthesize nanostructured magnesium hydrides, such as mechanical milling, chemical reduction, vapor deposition, and hydrogenation method. The hydrides were formed as particles (sphere, cube, rod, octahedron, *etc.*), hollow particles, thin films, and porous matrixes. de Jong *et al.* systematically studied the surface-induced destabilization effect of MgH_2_ grains using density-functional theory (DFT) calculations [215]. The results indicated the hydrogen desorption energy decreases significantly when the MgH_2_ crystal grain size is smaller than 1.3 nm. The decomposition temperature of an MgH_2_ crystallite with 0.9 nm size became 200°C. The surface-induced destabilization effect of MgH_2_ grains was further experimentally studied and supported by a report from Buckley's group (Figure 13) [216]. It was found that the MgH_2_ crystallite with 7 nm size exhibits decrease decomposition reaction enthalpy (nearly 4%) and reaction entropy (nearly 3%), although the decrement was not as high as the estimated simulation values. Fichtner and coworkers reported that the decrement of MgH_2_ crystallite size to 3 nm could offer lower decomposition reaction enthalpy (nearly 14%) and reaction entropy (nearly 12%) in comparison with those of bulk MgH_2_ [217]. Aguey-Zinsou and Ares-Fernández used tetrabutylammonium bromide as the surfactant to synthesize the surfactant-stabilized Mg nanoparticles with a diameter of 5 nm. The nanoparticle achieved hydrogen absorption and dehydrogenation at near room temperature (60°C and 85°C, respectively), which was significantly lower than those of bulk MgH_2_ (nearly 300°C) [218]. Urban and coworkers reported a moisture- and oxygen-stable crystalline Mg nanocrystals (about 4 nm)-poly(methyl methacrylate) composites. The composite demonstrated rapid hydrogenation at 200°C and 35 bar and achieved saturated concentration at 30 min of 6.0 wt% (in Mg, and 4.0% overall) in the absence of heavy-metal catalysts [219]. Prieto *et al.* also reported the decrement of activation energies of hydrogen absorption (115–122 kJ mol^–1^) and desorption (126–160 kJ mol^–1^) for MgH_2_ nanoparticles (25–38 nm diameter), as shown in Figure 14 [220]. Chen *et al.* employed DFT calculation to support the decrement of desorption enthalpies from 75 kJ mol^–1^ H_2_ for bulk MgH_2_ to 34.54 and 61.86 kJ mol^–1^ H_2_ for the nanowires MgH_2_ (diameters = 0.85 and 1.24 nm, respectively) [221]. These studies indicated the size reduction to nanoscale could decrease reaction entropy as well as the dehydration temperature or pressure.

Nanoscale hydrides prepared by direct physically or chemically synthesis suffer from the hydrogen capacity loss during the recycling of hydrides. This is because of the particle movement and agglomeration, and finally, nanoarchitecture collapse. As an effective solution to this problem, nanoconfinement hydrides expose cyclic sustainability to the hydrogen storage performance, along with conspicuous improvement of the kinetics and thermodynamics of hydrogenation/dehydrogenation properties. With the support of stable and rigid structural nanomaterials, the obtained nanoconfinement hydrides with enhanced mechanical stability to maintain the well-defined porous nanostructures.

**Figure 15:**
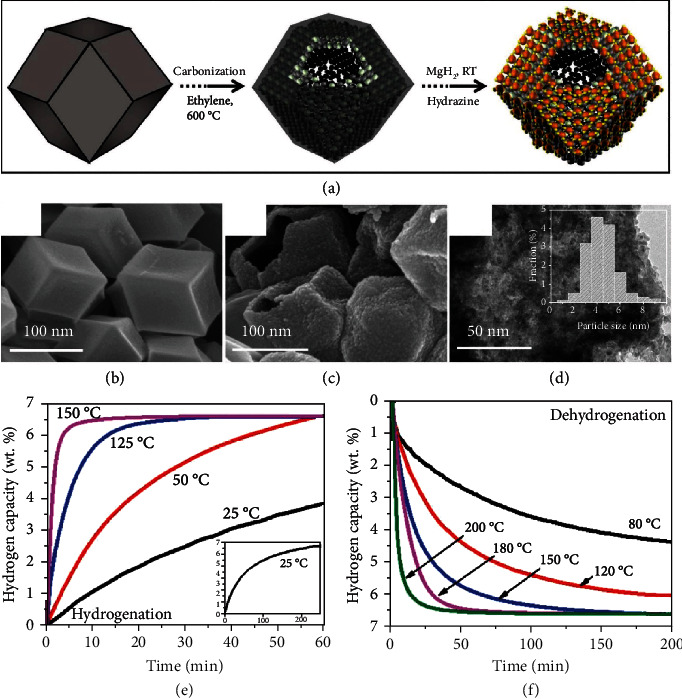
(a) Preparation of the self-assembled MgH_2_ on three-dimensional (3D) metal interacted carbon. (b) SEM images of metal interacted carbon. (c) SEM and (d) TEM images of the MgH_2_ embedded hollow 3D architecture of carbon (MHCH). The inset (d) shows the histogram distribution of MHCH size distributions. (e) Hydrogen absorption (at 10 bar) and (f) desorption (at 0.01 bar) of the MHCH at different temperatures. The inset (e) shows the hydrogen absorption of the MHCH at 25°C for 250 h. Reproduced with permission [222]. Copyright 2017, Royal Society of Chemistry.

Lee *et al.* synthesized a series of air-stable MgH_2_ nanoparticles embedded in 3D AC with periodic synchronization of transition metals (Figure 15) [222]. The high surface area, homogeneous distribution, and nanostructure (5.5 nm diameter) enable a high hydrogen storage density of 6.63 wt% and superior hydrogenation/dehydrogenation thermodynamics and kinetics. The MgH_2_ nanoparticles exhibited rapid hydrogenation kinetics at 180°C and 10 bar within 5 min and a dehydrogenation condition at 180°C and 10 bar for over 100 cycles, emphasizing their cycling stability and practicality. Recently, a novel and facile solid-state method was used to prepare MgH_2_ nanoparticle-graphene nanosheet composites for hydrogen storage [223]. The MgH_2_ composites exhibited improved hydrogen storage properties with hydrogenation temperature and pressure at 250°C and 20 bar. Under 325°C and vacuum conditions, the MgH_2_ composite could rapidly release 5.1 wt% hydrogen in 20 min. In addition to nanoparticles, Chen and coworkers fabricated MgH_2_ nanowires with diameters of 30–170 nm through chemical vapor deposition (CVD) [224]. The results indicated the nanowires with small diameters of 30–50 nm carrying superior hydrogen absorption/desorption kinetics and capability. Such nanowires exhibited a hydrogen intake capability of 7.6 wt% at 200°C and 4-20 bar, and the dehydrogenation was carried out at 200°C and 0.2–6 bar. The nanostructured MgH_2_ demonstrates significant advantages for hydrogen storage applications. Nevertheless, MgH_2_ nanoparticles are generally oxygen- and moisture-sensitive and with poor mechanical properties. The research on providing stable MgH_2_ materials with rapid hydrogen releasing and strong mechanical properties is of interest nowadays.

Aguey-Zinsou *et al.* used CNTs as a template for the formation of nanoscale hydrides (*i.e.*, NaAlH_4_, LiAlH_4_, and LiBH_4_ nanoparticles) [225]. The resulting confinements present a profound impact on the hydrides desorption properties. The activation energy of the H_2_ release from these hydrides was significantly diminished: approximately 45 kJ/mol and ~88 kJ/mol for NaAlH_4_-CNT and LiBH_4_–CNT, respectively, which much lower than that for their bulk counterparts (*i.e.*, bulk NaAlH_4_ (120 kJ/mol) and bulk LiBH_4_ (146 kJ/mol)). Besides, the reaction pathway of the dehydrogenation process was changed. For the bulk LiAlH_4_, two dehydrogenation steps with the activation energy at 82−115 kJ/mol and 86−90 kJ/mol, respectively. While only one single step with activation energy at around 64 kJ/mol was observed for LiAlH_4_-CNT. LiBH_4_ modified by SBA-15 (mesoporous silica) to produce LiBH_4_/SBA-15 nanocomposites underwent rapid hydrogen release at about 100°C, which significantly lowered than bulk LiBH_4_ (above 300°C). The onset dehydrogenation temperature of the confined LiBH_4_ decreased to 45°C. Furthermore, the LiBH_4_/SBA-15 nanocomposite can release around 8.5 wt% hydrogen within 10 min at 105°C [226]. The study of the NaZn(BH_4_)_3_/SBA-15 demonstrated that the dehydrogenation rate of the space-confined NaZn(BH_4_)_3_ is significantly improved (5.7 wt% hydrogen released in 90 min) and a low hydrogen release temperatures ranging from 50 to 150°C [227]. NaAlH_4_ confined within MOF-74(Mg) ensure reversible and low-temperature hydrogen storage [228]. The nano-NaAlH_4_@MOF-74(Mg) composite displays the first H_2_ desorption temperature around 50°C much lower than that of bulk sodium alanate (150°C). Plus, the activation energy for H_2_ release decreases from 79.5 kJ/mol for bulk Ti-doped NaAlH_4_ to 57.4 kJ/mol for nanoconfined NaAlH_4_.

**Figure 16:**
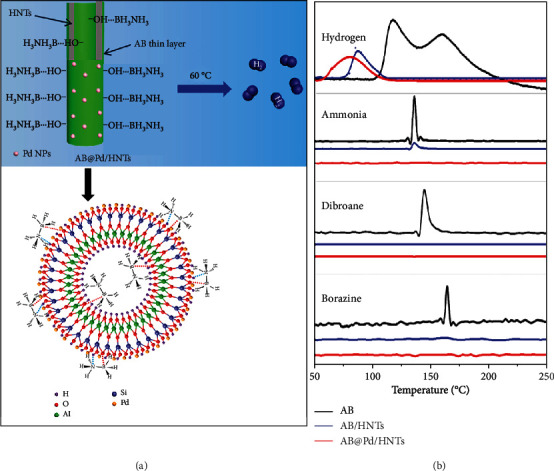
(a) The schematic representation of AB confined into the Pd/HNTs to generate H_2_ at 60°C. (b) MS profiles of AB, AB/HNTs, and AB@Pd/HNTs. Reproduced with permission [231]. Copyright 2020, American Chemical Society.

Through intelligent confining AB into nanomaterials, its overall hydrogen storage properties are significantly improved, facilitate its application in energy distribution and mobile platforms [30, 229–235]. A nanocomposite (AB/SBA-15) prepared by coating AB within a mesoporus silica SBA-15 exhibited the reduced onset temperature for H_2_ release and an improved dehydrogenation rate (half-life for hydrogen release is 85 min at 50°C) [229]. The barrier for H_2_ release from the AB/SBA-15 nanocomposite significantly decreased (Ea ~67 kJ/mol) than pure AB (Ea ~200 kJ/mol). Furthermore, the dehydrogenation of AB in the scaffold releases considerably less heat (enthalpy ~-1 kJ/mol) than that in the pure AB (enthalpy ~-21 kJ/mol). It means that the reverse hydrogenation reaction to store H_2_ into AB/SBA-15 would much easier than neat AB and enhance the (de)hydrogenation reaction reversibility. Monodisperse MnO_2_ hollow spheres (MHS) act as scaffold to mix with AB yielded MHS/AB composite with enhanced thermal dehydrogenation properties (the first dehydrogenation temperature is around 60°C) [230]. Besides, the generation of the volatile by-products was completely inhibited as well. Encapsulation of AB into Pd/natural halloysite nanotubes (HNTs) was prepared by Zhang *et al.* (Figure 16) [231]. The initiation temperature of H_2_ evolution for the obtained AB@Pd/HNTs is 60°C, low Ea 46 kJ/mol, and the generation of volatile by-products (*e.g.*, ammonia, diborane, and borazine) was depressed significantly.

**Figure 17:**
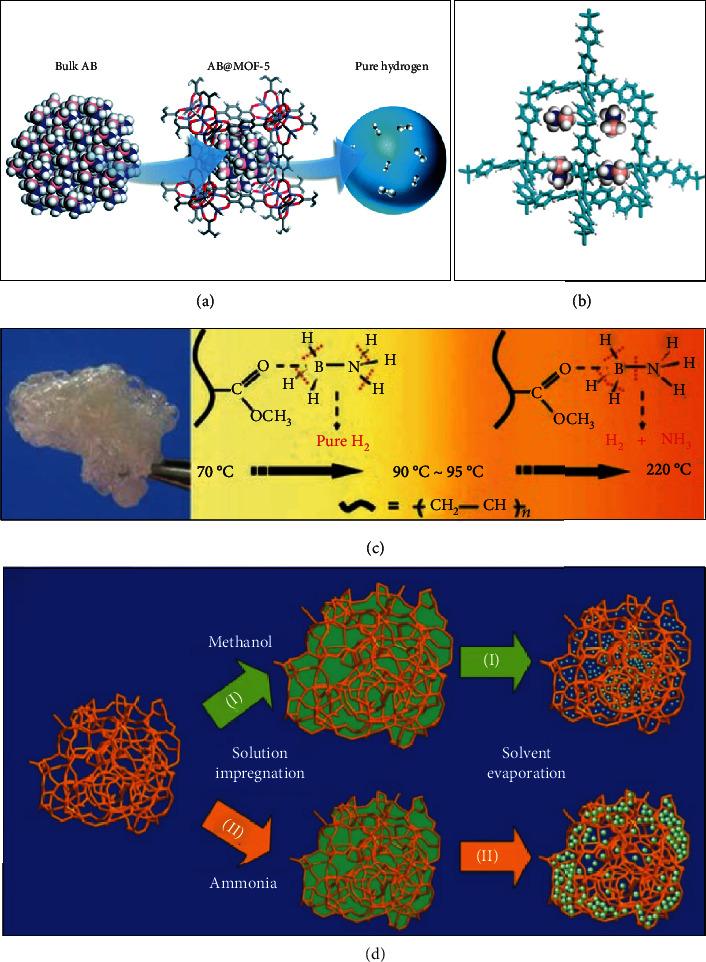
(a) AB@MOF-5 nanocomposite [232]. (b) AB@PAF-1 nanocomposite [235]. (c) PMA-AB polymeric nanocomposite and its proposed thermolysis mechanism [233]. (d) The preparation of AB-PSDB polymeric nanocomposite [234]. (e) Reproduced with permission [232]. Copyright 2014, Royal Society of Chemistry. Reproduced with permission [235]. Copyright 2012, American Chemical Society. Reproduced with permission [233]. Copyright 2010, Wiley-VCH. Reproduced with permission [234]. Copyright 2012, Royal Society of Chemistry.

Due to the ordered crystalline lattice structure and a high degree of synthetic flexibility to specially tailor the nanostructure, MOFs represent powerful scaffolds to support hydrides for hydrogen storage (Figure 17) [30, 232]. AB nanoconfinement in MOF-5 (AB@MOF-5) was found to dramatically diminish its hydrogen desorption temperature to 55°C and decrease its hydrogen desorption activation energy to 64.3 kJ/mol (Figure 17(a)) [232]. During the pyrolysis of AB@MOF-5, the by-product (ammonia) is produced together with H_2_. To eliminate the ammonia generation, a MOF-confined AB system (AB@JUC-32-Y) is developed [30]. AB@JUC-32-Y significantly reduced the onset H_2_ release temperature to 50°C. And the H_2_ release rate of AB inside the scaffold improved to completely release (∼13 wt% hydrogen) at 95°C within 3 h. Besides, the MOF-confined AB system effetely avoided the formation of ammonia to prevent the catalyst poison of PEMFC. Organic polymers are another alluring scaffold materials for chemical hydrides [233–235]. Poly(methyl acrylate)-confined AB (PMA-AB) was obtained by the solution-blending method. Due to the carbonyl group in the polymer, the material exhibited low dehydrogenation temperature (begin from 70°C) developed (Figure 17(c)) [233]. Also, AB molecules confined in hypercrosslinked porous poly(styrene-*co*-divinylbenzene) resin (AB-PSDB) to achieve remarkably improved kinetic and thermodynamic properties (about 8.5 wt% hydrogen desorption within 2 h at 80°C, released ~8 wt% H_2_ in 20 min and 11 wt% within 2 h at 100°C) developed (Figure 17(d)) [234]. The first decomposition temperature is reduced to about 50°C. A low-density and highly porous aromatic framework (PAF-1) is also utilized to support AB to obtain AB@PAF-1 composite developed (Figure 17(b)) [235]. The first decomposition temperature of AB in the PAF-1 is around 50°C in the absence of any volatile by-products. Thus, the brilliant integration of nanotechnology and hydrides exhibits excellent hydrogen ad(de)sorption properties, which is the ongoing and future research direction.

Nanomaterial-based hydrogen storage technologies have aroused increasing attention in hydrogen sustainable development. Exploiting the ultrafine nanostructures, nanomaterials offer new insights and opportunities for hydrogen storage. As the molecular hydrogen storage via physisorption using nanomaterial adsorbents is a surface phenomenon, the hydrogen storage capacity is in line with the increase of the material surface area. Therefore, those porous nanomaterial adsorbents, involving nanoporpus carbon materials, MOFs, COFs, PAFs, and nanoporous organic polymers, with a high surface area exhibit excellent storage ability. Of these, MOFs with high crystallinity, large surface area, and stability are a class of outstanding adsorbents, displaying great potential in hydrogen storage. For example, the hydrogen storage capacity of MOMs-399 with a particularly high BET area 7157 m^2^/g reaches hydrogen uptake capacity 9.02 wt% (45 bar, 77 K) [116], NU-100 has a BET area 6143 m^2^/g and a hydrogen uptake capacity 10.0 wt% (56 bar, 77 K) [52], and MOF-210 gives the highest capacities of hydrogen storage in MOFs which researches as high as 17.6 wt% (80 bar, 77 K) [28].

However, due to the weak attraction force, the stored H_2_ via pure physisorption is able to release from nanoadsorbents triggered by thermal. Because of the low H_2_ physical sorption enthalpy, cryogenic temperatures (around -196°C) and/or high pressures are required for the adsorption. Therefore, to maintain such a low temperature, the thermal insulation equipment is necessary, which resulting in energy-consuming, overall-cost-raising, and limitations for transportation and onboard storage. Moreover, hydrogen adsorption is an exothermic process, which brings additional technical problems. Consequently, future research direction for hydrogen physical adsorption should focus on the development of hydrogen reversible storage at room temperature to satisfy the practical interest. Strategies, such as increasing the surface area, enhancing the binding force by employing metals or other functional groups, and combining with other storage approaches (*i.e.*, encapsulation of hydrogen-rich molecules into nanomaterials), are worth trying. Also, seeking new structures with higher superficial areas and free volume and/or optimizing the nanostructure, composition, and preparation methods to tune the porous nanostructures to alter the hydrogen storage properties are promising.

Another highly promising hydrogen storage approach based on nanomaterials is chemisorption, in which, the atomic hydrogen chemically bonded with the metal or other element. The strong chemical bond in hydrides results in sluggish kinetics and unacceptable hydrogen desorption temperature, reduce their efficiency and suitability in mobile applications. Applying nanotechnology in the fabrication of catalysts and/or hydrides emerges as a rational strategy to significantly improve the hydrogen ab(de)sorption properties. The catalyst/nanomaterial nanocomposites display a compelling increase of the interaction surface area between the catalysts and hydrides and provide tremendous opportunities for the modulation of their electronic structures, leading to the improvement of the catalytic activity and efficiency as well as recyclability. Through the permutation and combination of suitable catalysts and nanomaterials, a desirable hydrogen storage medium with outstanding storage performance can be attained. In contrast to pure hydrides, nanoscale hydrides process a new nanoarchitecture with a higher surface area, additional hydrogen reaction sites, and shorter diffusion distances to conspicuous enhance the kinetics and thermodynamics of hydrogen ab(de)sorption properties [30, 228, 231, 247]. For instance, the hydrogen ab(de)sorption process can be conducted at near room temperature (below 85°C) with a rapid reaction rate using nanoscale hydrides to meet the practical interests. Moreover, exploiting nanoscaffold to support the hydrides can achieve good reversibility with minimal capacity loss during the ab(de)sorption cycling of hydrides, although this will reduce the hydrogen capacity (as the overall material weight increased). A feasible solution is the adoption of nanomaterials featuring ultrahigh surface area to increase the hydride loading content to finally improve the hydrogen content. Besides, the incorporation of lightweight elements (such as Li, Be, and Mg) into the material is another opportunity. Accordingly, the nanoscale hydrides present a considerable promising strategy for hydrogen storage toward actual interests and could be used firstly in practical applications.

## 3. Applications of Hydrogen Storage Systems and Future Perspectives

The adoption of hydrogen as an alternative fuel can be envisaged in the fields of stationary energy storage, hydrogen logistics, and onboard hydrogen generation within mobile applications. Conventionally, hydrogen physically stores as compressed gas or cryogenic liquid. They are the initial commercialized storage approach in both stationary and mobile applications, but low hydrogen capacity, high cost, and safety issues hamper their future long-term development. Applying nanomaterials in solid hydrogen storage, involving molecular hydrogen physical adsorption, atomic hydrogen chemical adsorption, and as functional supporting materials for hydrides, provides pronounced benefits. They are safe, high hydrogen content, and more importantly, the storage performance can be synthetically tailored, are future research directions for hydrogen-powered light duty vehicular applications.

### 3.1. Hydrogen Fuel Cells

**Figure 18:**
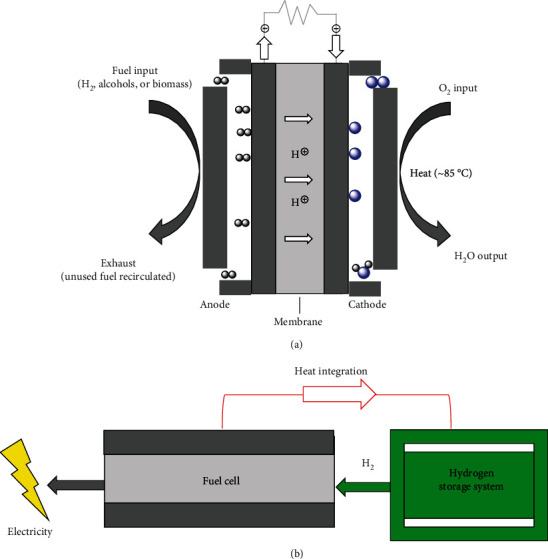
(a) General schematic of a polymer electrolyte membrane fuel cell. (b) Thermal integration between the hydrogen storage system and the fuel cell.

Owing to the low efficiency, the direct consumption of hydrogen in internal combustion engines becomes less popular. Instead, the employment of hydrogen energy in fuel cells has brought widespread awareness (Table 8) [248–250]. Fuel cells are electricity-generating devices, in which the chemical energy of hydrogen directly and efficiently convert into electrical energy with water as the only reaction product and zero greenhouse gas emission (Figure 18(a)) [251, 252]. In a typical hydrogen fuel cell, hydrogen and oxygen continuously flow to the anode and cathode, respectively, giving the electrochemical reactions to produce an electric current [248].

Based on the electrolyte used, fuel cells can be classified into six species [253], which are polymer electrolyte membrane fuel cell (PEMFC) (Figure 18(a)) [253–257], direct methanol fuel cell (DMFC) [258–260], alkaline fuel cell (AFC) [261–263], phosphoric acid methanol fuel cell (PAFC) [236, 237], molten carbonate fuel cell (MCFC) [264–266], and solid oxide fuel cell (SOFC) [267, 268]. Additionally, the reversible fuel cell (RFC) [269–271] attracts great attention, taking advantage of its ability to produce electricity as well as store excess energy in the form of hydrogen.

PEMFC, or also term proton exchange membrane fuel cell, is the desirable prospect for hydrogen-powered cars, portable or micropower systems, and transportation to power stations for further applications [248, 249, 253–257]. It uses a proton exchange membrane as an electrolyte and porous carbon electrodes catalyzing by a platinum or platinum alloy. PEMFC contains high power density, relatively low operation temperature (around 85°C), good durability, rapid change in power on demand, promising power-to-weight ratio, and fast start-up and shutdown. However, the PEMFC catalysts are sensitive to some volatile gases (*e.g.*, carbon monoxide and ammonia) and easy to get poison to cause performance degradation. The DMFC is similar to the PEMFC, which uses a polymer membrane as an electrolyte. But DMFC is powered by pure methanol instead of hydrogen and can realize relatively large-scale fuel storage as the higher energy density of methanol than hydrogen. DMFCs are frequently employed to produce electricity for portable power applications such as cell phones or laptop computers [258–260]. AFC utilizes an aqueous solution of potassium as the electrolyte and is the most initial fuel cell widely adopted in the submarines and space crafts. Due to the rapid rate of the electrochemical reactions, AFC demonstrates efficiencies above 60% in space employment. Whereas, AFC is sensitive to CO_2_, even a small amount of CO_2_ in the air can destroy its operation and endurance as the formation of carbonate [261–263]. Liquid phosphoric acid is used as an electrolyte in PAFC, which is the most mature cell type. Due to the large electrical power production ability (>50 kW), PAFC is typically used for stationary power generation for commercial premises, and power engendering for large-sized transportation media (*i.e.*, buses and locomotives) as well. But, PAFCs are large, heavy-weight, and expensive [236, 237]. The MCFC uses a molten carbonate salt as the electrolyte. It has the potential to be fueled with coal-derived fuel gases, methane, or natural gas for electrical generation in manufacturing and martial applications. MCFCs operate at high temperature (650°C); the fuel is converted to hydrogen within the fuel cell itself by the internal reforming process. By coupling with a turbine, the efficiency of MCFC can reach approximately 65%, and overall fuel use efficiency could top 85% when the waste heat is captured and employed. While the high-temperature operation of MCFC significantly diminishes its durability [264–266]. Compared to MCFCs, SOFCs run at an even higher temperature (800-1000°C) and utilize a hard, nonporous ceramic as the electrolyte. Similar to MCFCs, SOFCs can be fed with natural gas, biogas, and coal-based gases as well. The efficiency of SOFCs is about 60%, and overall fuel efficiency could approach 85 %. In addition, SOFC is the most sulfur-insensitive cell and also can resist the impact of carbon monoxide. However, slow start-up and nondurability caused by the high-temperature operation of SOFCs restrict their application in transportation [267, 268].

The heat required for H_2_ release from storage systems can be provided via three ways: (1) using electrical heating by sharing the electricity generated by fuel cells, (2) applying a hydrogen burner to supply heat, and (3) heat combining between the endothermic H_2_ release process and waste heat generated by the operation of fuel cells. Compared to the first two methods, the third option is more applicable for real application to reach the most energy-efficient at an acceptable cost. Figure 18(b) demonstrates a schematic design on the heat combination between the fuel cell and hydrogen storage systems. The hydrogen storage system affords fuel for the fuel cell to directly generate electricity, while the yielded waste heat can, in turn, drive H_2_ release. Accordingly, integrating of suitable species of fuel cells and hydrogen storage systems, highly hydrogen energy efficiency is achievable [272].

### 3.2. Stationary and Mobile Applications

Hydrogen energy is extensively employed in stationary applications, involving large hydrogen power stations, hydrogen refilling stations, and industrial utilizations. In a stationary application, the relatively harsh hydrogen storage condition is comparatively bearable. For example, the massive storage systems, relatively high hydrogen release temperature and pressure, and sluggish hydrogen uptake/release rate are acceptable in a hydrogen power station. As aforementioned, compressed hydrogen gas can be stored in different containers. Among these, the salt caverns are capable to store large-scale compressed gas for seasons for further applications, such as in the chemical industry and salt industry. The salt domes present an alluring opportunity for compressed hydrogen gas storage with designable hydrogen injection/withdrawal pressure, adaptable cavern volume and depth, safety, and long-term reactivity. It is estimated that Europe with an overall technical salt domes hydrogen storage capacity around 84.8 PWh_H2_, in which, Germany with 9.4 PWh_H2_ represents the highest national hydrogen storage potential [33, 34]. In Romanis, a project named HyUnder is also aimed at storing a large amount of H_2_ underground in salt caverns [14, 15]. Other nations, such as the USA, the UK, and China are also implemented salt caverns for hydrogen storage [33, 34]. However, regional limitations are too strong for the salt cavern to become global availability.

**Figure 19:**
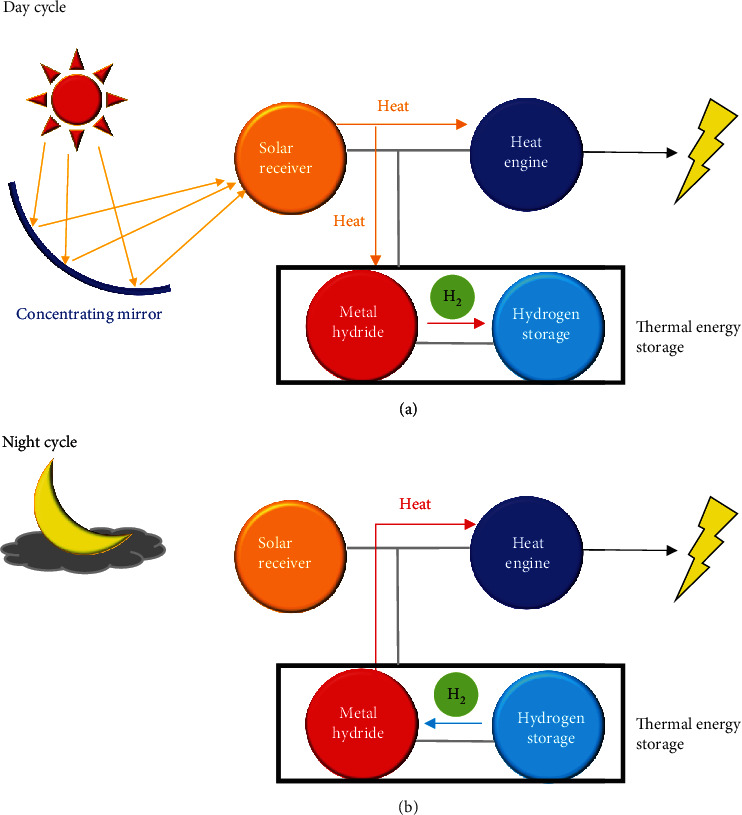
Schematic diagram of the operation of a CSP system during the (a) daytime and (b) night time.

The concentrating solar thermal power (CSP) contains a thermal energy storage (TES) system that can be used to produce electrical energy using sunlight radiation (Figure 19) [46, 273–277]. The CSP technology is low cost and eco-friendly and can generate energy for self-supply. In a typical run, the CSP plant provides power for electricity generators and the TES system at sunlight rich time and requires the TES to produce electrical energy during the night or cloud cover times. Currently, molten nitrate salts are widely used as TES media to gather solar energy. Although they are economical-attractive, a huge amount of molten nitrate salts is needed to generate enough energy on demand since their low energy density. Taking advantage of the high energy density of high-temperature metal hydrides (MH_T_high_), they have been promoted as new TES media to replace molten nitrate salts recently. In this TES system [46], under the sunlight, MH_T_high_ (such as MgH_2_, Mg_2_NiH_4_, and LiH) with large ab(de)sorption enthalpy is first heated by solar energy to store thermal energy and release H_2_ which stored into the low-temperature metal hydride (MH_T_low_, such as LaNi_5_H_6_, FeTiH_2_, and TiV_2_H_4_) with relatively low ab(de)sorption enthalpy. The reversed reaction (H_2_ released from MH_T_low_ into MH_T_high_) is used to generate energy during the night. The reformation of high-temperature metal hydride releases exhaust heat for electricity production in the steam turbo generator. The use of MH_T_low_ can provide H_2_ to MH_T_high_ to generate heat, while it requires heat management and increases the overall cost. To overcome these problems, Sheppard *et al.* used the compressor hydrogen gas (in the underground salt domes or lines rock caverns) to supplant MH_T_low_ to supply H_2_ to MH_T_high_ [277]. The overall installed cost of the combination of MH_T_high_ and compressed H_2_ system is around US13.7 to US26.7/kWh, which is cheaper than the molten nitrate salt system. Furthermore, optimizing the reaction condition of MH_T_high_ is an effective way to increase the work efficiency and reduce the cost. The CSP technology is extensively used for stationary electricity generating; however, the large space plant and slow kinetics restrict its mobile applications.

**Figure 20:**
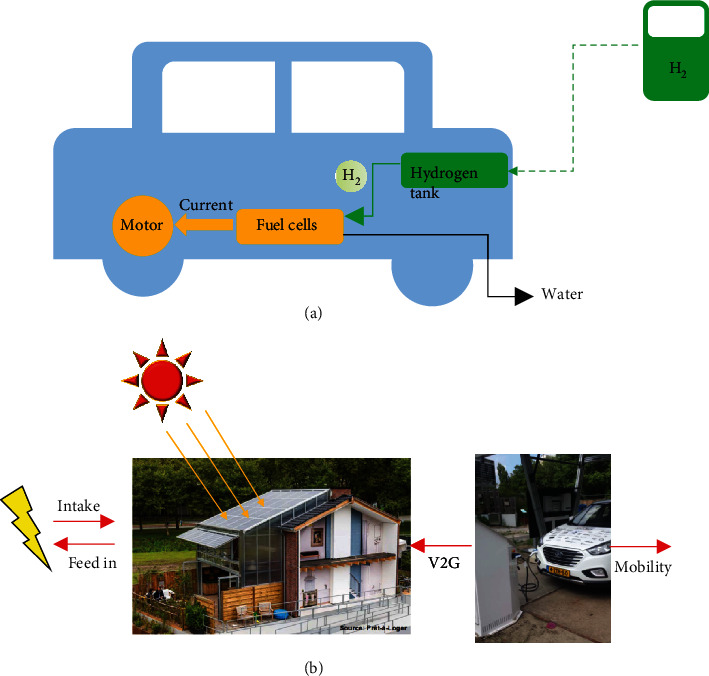
(a) Fuel cell vehicle with onboard storage. (b) Schematic representation of the hybrid system for a net-zero-energy residential environment. The arrows represent the energy flows between the components [284]. Reproduced with permission [284]. Copyright 2018, Elsevier.

Hydrogen energy in mobile applications plays a key role in the thriving of a hydrogen economy. It involves hydrogen logistics and hydrogen-powered mobile platforms (such as automobiles, ships, locomotives, and portable power devices). The hydrogen distributed worldwide on demand is important as the hydrogen energy-rich region is different from those with the high demand. In contrast to the stationary power supplication, hydrogen fuel applied in mobile platforms has more critical requirements, concerning weight, volume, cycling life, and kinetics and thermodynamics properties of hydrogen uptake and release. Among all the hydrogen generation mobile platforms, hydrogen fuel cell vehicles (HFCVs) are the most widely studied. The widespread of HFCVs may significantly contribute to the air quality, environment, and climate. As discussed earlier, PEMFC is the most fitting candidate that meets the requirements of hydrogen-fueled cars. The structure of a fuel cell automobile with hydrogen onboard storage is illustrated in Figure 20(a) [278, 279]. Hydrogen can be stored in different formations in a fuel tank and released in a controlled manner. The liberated H_2_ serves as the fuel to PEMFC, in which H_2_ directly converts to electricity for car operation. On the other hand, the thermal caused by the operation of PEMFC (around 85°C) can be appropriated for the endothermal H_2_ release.

Some car manufactures including Toyota, Honda, Mercedes-Benz, and Hyundai have launched fuel cell vehicles equipped with the compressed H_2_ fuel tank [280–283]. The pressured gas storage system is able to contribute 5.2 and 5.5 wt% H_2_ at 700 and 350 bar, respectively. Assuming 50% PEMFC efficiency, 5.6 kg of useable H_2_ is required for around 300-mile driving distance [15]. Hence, the compressed gas storage system could approach the near-term driving range target. However, this storage method is unable to reach the ultimate DOE requirement (6.5 wt%), and the high cost of the CFRP tank and safety issues is practical obstacles to hamper its extensive application. Consequently, developing new efficient, high energy density, economically acceptable, and safe storage systems is essential for the hydrogen economy.

An ideal onboard hydrogen storage system in the fuel cell vehicles needs the following: (1) a medium with light-weight, compact, and high hydrogen capacity (material capacity ≥10 wt% or system capacity ≥6.5 wt%), (2) liberation the hydrogen at a temperature approaching the PEMFC operation temperature (∼85°C) to achieve efficient heat coupling, (3) increase the H_2_ release kinetics to sustainable supply hydrogen on demand (up to 2 g H_2_ s^−1^) and fast the refueled rate (∼15 g H_2_ s^−1^) to satisfy the requirement for the normal execution, (4) improve the reversibility of (de)hydrogenation reaction for easily handling and economically visualization, and (5) eliminate the production of volatile by-products (such as CO_2_ and ammonia) to avoid the poison of PEMFC catalysts to increase sustainability and duality. Besides, the overall cost, convenience, and security issues should meet the actual interest. It is impartial to say that none of the known hydrogen storage systems discussed in the review can fulfill all of these desirable properties to the full extent. A temporary feasible option for HFCVs is the irreversible off-board regeneration strategy. It can alleviate obstacles associated with the current reversible onboard storage approaches and can be applied for the portable power platforms (such as batteries, sensors, and switchable devices). However, the off-board regeneration method needed to replace the entire empty fuel tank with a fresh one, which is inconvenience, cost-unattractive, and energy-consuming. Thus, for vehicular applications, the reversible onboard hydrogen storage system with an excellent storage performance is desired for long-term development.

In a typical HFCV, the electrical energy generated from hydrogen energy is mainly for transport, whereas, most vehicles are parked for around 95% of their lifetime. The new concept is to offer vehicle-to-grid (V2G) [284–287] services to attain the integration of mobility and energy generation system (Figure 20(b)). During the power outage period of the house, the HFCV generates electricity to support the residential operation. It is estimated that using a power output socket, the HFCV could deliver up to 10 kW direct current to the alternating current grid by a grid-tie inverter. Renewable sources, such as solar and wind, are well established but suffer from intermittent nature, while hydrogen is inexhaustible but still under development. Via the intelligent combination of renewable technologies with different features, the prevailing associated problems (such as energy efficiency and environment issues) can be mitigated and a net-zero-energy residential environment can be achieved (Figure 20(b)).

## 4. Conclusions and Future Perspectives

The widespread of hydrogen fuel has a profound effect for the expected transition from a fossil fuel-based system to a clean energy system. This dramatically reduces the emission of greenhouse gases to improve the environment and climate alternation, as well as relieves the energy crisis caused by the fossil fuel depletion using an inexhaustible fuel source to meet the energy demand. Hydrogen fed into fuel cells to be directly converted to electricity for stationary, transportation, and onboard mobile applications. Furthermore, the thermal integration between the fuel cell heat-generating and hydrogen release heat-consuming processes is an effective way to further improve energy efficiency. One of the key disadvantages of hydrogen energy comes from its low density, which results in the hassle for its energetically efficient storage. Hydrogen energy could represent the dominating future energy carrier if the bottleneck is overcome.

The conventional hydrogen storage system features physically increase hydrogen gas density by pressure or cryogenics, suffers from low hydrogen capacity, high cost, and safety issues. Hydrogen storage systems based on nanomaterials are highly attractive alternatives. The hydrogen storage based on solid media with high energy density, safe, and some metal hydrides with good reversibility demonstrates great potential for automobile applications. Currently, most solid hydrogen storage systems are completed by the physisorption of molecular hydrogen via nanoporous materials. These nanomaterial absorbents display the high hydrogen content absorption and the easy-handling desorption. Whereas, the low temperatures storage condition needed to be improved to facilitate its mobile applications. Solutions such as an increase in the surface area of nanomaterials and introduce functional sites to improve the storage condition are ongoing efforts. Hydrogen chemically bounded into hydrides with high hydrogen content presents another interesting solid hydrogen storage method. However, the unfavorable kinetics and thermodynamics properties severely decrease their potential in onboard hydrogen storage. A diligent strategy is to complement advantages of nanotechnology and hydrides to prepare nanoscale hydrides, which demonstrate the significantly different nanostructures to positively alter the hydrogen ab(de)sorption properties.

Moreover, advances in the skeleton design and synthetic methods offer precise and effective routes to develop functional nanoporous materials. The control of crystal growth, morphology, defect sites, and the stacking layers are important for the crystalline materials, *i.e.*, MOFs, COFs, PAFs, and hydrides, with high hydrogen storage capabilities. One of the application limitations of the crystalline materials is their poor processability. The development of composites and membranes hybridizing MOFs or COFs with soft materials such as polymers is beneficial for practical use [44, 288]. Recently, 2D MOF, COF, and hydride nanosheets are attracting increasing attention [289, 290]. 2D nanosheets is advantageous on their lightweight, high specific surface and flexibility, they could be expected to be desirable nanomaterials for hydrogen storage. Machine learning has recently been applied to accelerate the design and synthesis of porous materials such as MOFs and COFs [291, 292]. Machine learning can not only use to understand the relationship between their structure and performance but also can simulate and optimize the synthetic feasibility, long-term stability, and hydrogen absorption/desorption mechanism. Machine learning would become a powerful tool to further enhance design and development of new hydrogen storage nanomaterials.

Despite the great fundamental improvement that has been achieved, future efforts are still necessitated to optimizing the existing technique and/or explore new medium with excellent hydrogen storage performance to achieve high energy efficiency and economically viable. This article systematically collated the state-of-art solid-state hydrogen storage systems; each system has its advantages and disadvantages. In terms of their characteristics and sustainable development demand, complementary advantages of different strategies may be the future research direction. For example, functionalizing nanomaterial with hydrogen-rich moieties to increase the hydrogen storage performance and adaptability for transportable purposes. Given the tremendous candidates and complexity associated with the hydrogen storage system, it is challenging to unveil the reaction rules and find out all the potential storage medium by relying solely on experimental methods. Theoretical simulations have become a powerful tool to support the mechanism study and investigate the new hydrogen storage system. Combination of hydrogen energy with current technique mature renewable energy (such as solar and wind) is arguably the best short term approach. In that case, electricity is efficiently generated without giving rise to any burden on the environment, sufficient energy can be afforded in correspond with society's demand, and the intermittent nature of solar and wind can be overcome.

In summary, significant key advancements have been achieved to date in hydrogen storage, offering tremendous opportunities for hydrogen-based fuel as the substitution of fossil-based fuels and will continue to contribute to sustainable development.

## Figures and Tables

**Table 1:** Summary of the DOE goals for hydrogen storage in onboard vehicular applications [14].

Storage parameter	Unit	2025	Ultimate
Storage capacities			
Gravimetric capacity			
Material-based gravimetric capacity	kWh/kg	1.8	2.5
System-based gravimetric capacity	kg H_2_/kg system	0.055	0.065
Volumetric capacity			
Material-based volumetric capacity	kWh/L	1.3	1.7
System-based volumetric capacity	kg H_2_/L	0.04	0.05
Storage system cost	$/kWh net ($/kg H_2_)	9 (300)	8 (266)
Durability/operability			
Operating ambient temperature	°C	-40/60 (sun)	-40/60 (sun)
Min/max delivery temperature	°C	-40/85	-40/85
Min/max delivery pressure	bar	5/12	5/12
Cycle life (uptake/release cycles)	cycles	1500	1500
System fill time (for 4-10 kg)	min	3-5	3-5
Fuel purity (H_2_ from storage)	%H_2_	99.97%	99.97%

**Table 2:** Estimated performance for conventional hydrogen storage approaches.

H_2_ Storage system	H_2_ density (g/L)	Gravimetric (kg H_2_/ kg system)	Volumetric (kg H_2_/L)
Compressed gas (350 bar, 300 K)	24	0.055	0.0185
Compressed gas (700 bar, 300 K)	40	0.052	0.0277
Cryogenic storage (1 bar, 20 K)	70	~0.05	
Cryocompressed (276 bar, 20 K)	87	0.058	0.043

**Table 3:** Different hydrogen absorption ways [46].

	Physisorption	Chemisorption	Kubas interaction
Binding enthalpy (kJ/mol)	-4–-10	-100–-200	-20–-70
Binding energy (eV)	0.04–0.1	2–4	0.1–0.8

**Table 4:** Experimentally measured hydrogen storage properties of selected nanoporous carbon materials.

Carbon material	Storage conditions Temp. (K)/Press. (bar)	BET surface area (m^2^ g^–1^)	Hydrogen capability (wt%)	Ref
AC (Maxsorb)	77/30	3306	5.70	[64]
AC (Maxsorb)	303/100	3306	0.67	[64]
AC (AX-21)	77/60	2745	10.80	[65]
AC (KOH-treated)	298/100	2800	0.85	[66]
AC (KOH-treated)	77/20	3190	7.08	[67]
AC (KOH-treated)	77/20	2770	6.20	[68]
AC (KOH-treated)	77/20	3000−3500	7.03	[69]
AC (KOH-treated)	77/19	687	2.14	[77]
AC (Pt-doped)	298/100	2033−3798	1.10	[73]
AC (Pd-doped)	298/80	2547	5.50	[74]
AC ((Ni-B)-doped)	77/1.0	976	1.80	[75]
SWCNT	133/0.4	–	5–10	[80]
CNT	273–295/1.0	290–800	≤1.0	[81, 82]
CNT (film)	298/10	–	8.0	[83]
MWCNT	298/148	–	6.3	[84]
CNT (Li-doped)	653/1.0	130 (specific)	20	[85]
CNT (K-doped)	343/1.0	130 (specific)	14	[85]
MWCNT (Ca-doped)	– (electrochemical)	–	0.3	[86]
MWCNT (Co-doped)	– (electrochemical)	–	1.05	[86]
MWCNT (Fe-doped)	– (electrochemical)	–	1.5	[86]
MWCNT (Ni-doped)	– (electrochemical)	–	0.75	[86]
MWCNT (Pd-doped)	– (electrochemical)	–	0.4	[86]
CNF	298/120	51	6.54	[90]
CNF (KOH-treated)	77/40	1500–1700	3.45	[92]
CNF (N-doped)	298/100	870 (specific)	2.0	[96]
CNF (Ni-doped)	298/100	1310	2.2	[97]

**Table 5:** Experimentally measured hydrogen storage properties of selected MOFs, COFs, and PAFs.

Framework	Storage conditions Temp. (K)/Press. (bar)	BET surface area (m^2^ g^–1^)	Hydrogen capability (wt%)	Ref
MOF-5	(a) 78/20 (b) 298/20	2500−3000	(a) 4.5 (b) 1.0	[112]
IRMOF-8	298/10	1801	2.0	[112]
MOF-177	(a) 78/70 (b) 298/100	4600	(a) 7.5 (b) 0.62	[113, 114]
NU-100	77/56	6143	10.0	[52]
NU-109	77/45	7010	8.30	[115, 116]
NU-110	(a) 77/45 (b) 298/180	7140	(a) 8.82 (b) 0.57	[115, 116]
MOF-399	(a) 77/56 (b) 298/140	7157	(a) 9.02 (b) 0.46	[116]
Cr-MIL-53	77/16	1020	3.1	[117]
Al-MIL-53	77/16	1026	3.8	[117]
Cu-MOF-5	(a) 77/65 (b) 298/65	1154	(a) 3.6 (b) 0.35	[118]
MOF-210	(a) 77/80 (b) 298/80	6240	(a) 17.6 (b) 2.7	[118]
Be-MOF	(a) 77/ 1.0 (b) 298/95	4030	(a) 1.6 (b) 2.3	[122]
COF-1	(a) 77/1.0 (b) 77/70	711	(a) 1.7 (b) 3.8	[135]
COF-5	(a) 77/1.0 (b) 77/80	1590	(a) 0.1 (b) 3.4	[135]
COF-102	(a) 77/1.0 (b) 77/100	3620	(a) 0.5 (b) 10.0	[135, 136]
COF-102-3	(a) 77/100 (b) 300/100	–	(a) 6.5 (b) 26.7	[139]
COF-105	(a) 77/1.0 (b) 77/80	3472	(a) 0.6 (b) 10.0	[134, 135]
COF-108	(a) 77/1.0 (b) 77/100	4210	(a) 0.9 (b)10.0	[131–133]
CTC-COF	77/1.1	1710	1.12	[140]
COF-105 (Li-doped)	298/100	–	6.84	[145]
COF-108 (Li-doped)	298/100	–	6.73	[145]
COF-340-CoCl_2_	298/250	7400	7.00	[146]
PAF-1	77/48	5600	7.0	[27]
PAF-3	77/60	2932	5.5	[149, 150]
PAF-4	77/60	2246	4.2	[149, 150]
PAF-1 (KOH-treated)	77/1.0	1320	3.06	[151]
PAF-1 (Li-doped)	77/1.2	–	10	[153]
PAF-4 (Li-doped)	77/100	5525	20.7	[152]
PAF-4 (Li-doped)	233/100	5525	4.9	[152]
PAF-Mg	233/100	4479 (Langmuir)	6.8	[154]
PAF-Ca	233/100	4479 (Langmuir)	6.4	[154]
PAF-324	298/100	5372 (specific)	6.32	[155]
PAF-334	298/100	–	16.03	[155]

**Table 6:** Experimentally measured hydrogen storage properties of selected HCPs, CMPs, and PIMs.

Polymer material	Storage conditions Temp. (K)/Press. (bar)	BET surface area (m^2^ g^–1^)	Hydrogen capability (wt%)	Ref
HCP (polystyrene)	77/1.2	1930	1.5	[29]
HCP (polystyrene)	77/15	2920	3.04	[164]
HCP (polyphenylene)	77/15	1904	3.68	[165]
HCP (polyaminobenzene)	(a) 77/1.2 (b) 273/90	384	0.97 0.22	[166]
HCP (polyphenylene-Pt)	298/19	1399	0.21	[167]
CMP (poly(arylene-ethynylene))	77/1.0	1018	1.4	[168]
CMP (NCMP-0)	(a) 77/1.13 (b) 77/1.13	1108	(a) 1.5 (b) 2.0	[171]
CMP (E1)	(a) 77/1.13 (b) 77/8	1213	(a) 1.33 (b) 2.66	[172]
CMP (EOF-6)	77/1.0	1380	1.29	[174]
CMP (PCZN-8)	77/1.0	1126	1.35	[175]
CMP (Li-Doped)	77/1.0	834	6.1	[177]
CMP (PTAT, Li-Doped)	(a) 77/1.0 (b) 273/1.0	304	(a) 7.3 (b) 0.32	[178]
PIM-1	(a) 77/1.0 (b) 77/10	760	(a) 1.04 (b) 1.44	[181]
PIM (STP-II)	77/1.0	1990	1.9	[184]
HATN-PIM	77/120	772	3.86	[185]
PIM (PAF mixture)	77/100	1197	4.1	[186]
PIM (AC (20 wt%) mixture)	77/100	1130	3.7	[54]

**Table 7:** Hydrogenation/dehydrogenation conditions, activation energy (Ea), and hydrogen capability of hydrides.

Storage media	Storage conditions Temp. (K)/Press. (bar)	*E*_*a*_ (kJ/mol)	Hydrogen capability (wt%)	Dehydrogenation conditions Temp. (K)/Press. (bar)	Ref
LiH	1183/1.0	181.2	12.7	–	[195]
MgH_2_	557/1.0	174 [236, 237]	7.6	553/0.9	[196, 197]
MgH_2_ (5 wt% V-doped)	473/10	119 [236, 237]	5.8	573/0.15	[189]
MgH_2_ (5 wt% Ni-doped)	473/10	75 [236, 237]	5.0	573 / 0.15	[189]
MgH_2_ (Ti-Nb-doped)	(a) 673/40 (b) 573/40	50.7	(a) 6.8 (b) 5.7	(a) 673/1.0 (b) 573/1.0	[238]
MgH_2_ (TiH_2_-doped)	573/20	16.4	4.6	573/0.01	[239]
MgH_2_ (Cr_2_O_3_-doped)	573/1–2	86 [236, 237]	6.4	573/1–2	[189]
MgH_2_ (Nb_2_O_5_-doped)	573/8.4	95	7.0	573/vacuum	[240]
MgFeH_6_	623/2.72	166	5.0	623/0.001	[241]
MgH_2_ (5 nm)	333/20	–	1.34	358/0.003	[218]
MgH_2_ (5 nm)	473/35	25_(abs)_/79_(des)_	6.0	473/vacuum	[219]
MgH_2_ (2.8 nm)	355/33	–	2.8	352/–	[242]
MgH_2_ (6.0 nm, graphene composite)	473/30	23_(abs)_/65_(des)_	5.4	473/0.01	[243]
MgH_2_ (5.5 nm, deposit on AC)	453/10	31_(abs)_/43_(des)_	6.63	453/0.01	[222]
MgH_2_ (3 nm, graphene composite)	523/20	118.9	5.6	598/vacuum	[223]
MgH_2_ (Carbon composite)	387/20	–	6.0	773/vacuum	[244]
MgH_2_ (≤27 nm, deposit on carbon aerogels)	628/50	–	3.1	628/vacuum	[245]
MgH_2_ (30–50 nm, nanowire)	573/4–20	33.5_(abs)_/38.8_(des)_	7.6	573/0.2–6	[224]
PdH_0.6_	298/0.02	71	0.56	–	[246]

**Table 8:** Different fuel cells [249].

Electrolyte	PEMFC	DMFC	AFC	PAFC	MCFC	SOFC
	Proton exchange membrane	Polymer membrane	Potassium hydroxide	Liquid phosphoric acid	Liquid molten carbonate	Ceramic
Operation temperature (°C)	85	60-130	60-90	200	650	800-1000
Efficiency (%)	40-60	40	45-60	35-40	45-60	50-65
Typical electrical power	≤250 kW	<10 kW	≤20 kW	>50 kW	>1 MW	>200 kW
Possible applications	Vehicles, small stationary	Portable power application	Submarines, space crafts	Power stations	Power stations	Power stations
